# Energy-Efficient Boarder Node Medium Access Control Protocol for Wireless Sensor Networks

**DOI:** 10.3390/s140305074

**Published:** 2014-03-12

**Authors:** Abdul Razaque, Khaled M. Elleithy

**Affiliations:** Computer Science and Engineering Department, University of Bridgeport, Bridgeport, CT 06604, USA; E-Mail: elleithy@bridgeport.edu

**Keywords:** design, experimentation, performance, algorithms, sensor node, hybrid MAC protocols, BN-MAC protocol, mobility, intelligent decision-making (IDM) model, automatic active and sleep (AAS) model, least-distance smart neighboring search (LDSNS), wireless sensor network (WSN)

## Abstract

This paper introduces the design, implementation, and performance analysis of the scalable and mobility-aware hybrid protocol named boarder node medium access control (BN-MAC) for wireless sensor networks (WSNs), which leverages the characteristics of scheduled and contention-based MAC protocols. Like contention-based MAC protocols, BN-MAC achieves high channel utilization, network adaptability under heavy traffic and mobility, and low latency and overhead. Like schedule-based MAC protocols, BN-MAC reduces idle listening time, emissions, and collision handling at low cost at one-hop neighbor nodes and achieves high channel utilization under heavy network loads. BN-MAC is particularly designed for region-wise WSNs. Each region is controlled by a boarder node (BN), which is of paramount importance. The BN coordinates with the remaining nodes within and beyond the region. Unlike other hybrid MAC protocols, BN-MAC incorporates three promising models that further reduce the energy consumption, idle listening time, overhearing, and congestion to improve the throughput and reduce the latency. One of the models used with BN-MAC is automatic active and sleep (AAS), which reduces the ideal listening time. When nodes finish their monitoring process, AAS lets them automatically go into the sleep state to avoid the idle listening state. Another model used in BN-MAC is the intelligent decision-making (IDM) model, which helps the nodes sense the nature of the environment. Based on the nature of the environment, the nodes decide whether to use the active or passive mode. This decision power of the nodes further reduces energy consumption because the nodes turn off the radio of the transceiver in the passive mode. The third model is the least-distance smart neighboring search (LDSNS), which determines the shortest efficient path to the one-hop neighbor and also provides cross-layering support to handle the mobility of the nodes. The BN-MAC also incorporates a semi-synchronous feature with a low duty cycle, which is advantageous for reducing the latency and energy consumption for several WSN application areas to improve the throughput. BN-MAC uses a unique window slot size to enhance the contention resolution issue for improved throughput. BN-MAC also prefers to communicate within a one-hop destination using Anycast, which maintains load balancing to maintain network reliability. BN-MAC is introduced with the goal of supporting four major application areas: monitoring and behavioral areas, controlling natural disasters, human-centric applications, and tracking mobility and static home automation devices from remote places. These application areas require a congestion-free mobility-supported MAC protocol to guarantee reliable data delivery. BN-MAC was evaluated using network simulator-2 (ns2) and compared with other hybrid MAC protocols, such as Zebra medium access control (Z-MAC), advertisement-based MAC (A-MAC), Speck-MAC, adaptive duty cycle SMAC (ADC-SMAC), and low-power real-time medium access control (LPR-MAC). The simulation results indicate that BN-MAC is a robust and energy-efficient protocol that outperforms other hybrid MAC protocols in the context of quality of service (QoS) parameters, such as energy consumption, latency, throughput, channel access time, successful delivery rate, coverage efficiency, and average duty cycle.

## 1. Introduction

Wireless sensor networks (WSNs) have become an increasingly popular research topic in recent years. WSNs have produced promising solutions for several applications, such as intrusion detection, target detection, industrial automation, environmental monitoring, surveillance and military systems, medical diagnosing systems, and tactical systems [1]. WSNs consist of small sensor nodes disseminated in a targeted area to monitor the events for collecting the data of interest. WSNs also experience many challenging problems, including large energy consumption, network scalability, mobility, coverage, and uniformity [2]. These problems affect the lifetime of the network, increase the latency, and reduce the throughput. The limited battery life and harsh operating conditions cause further complications, which can lead to node failure [3]. Although significant research has been conducted on WSNs to maintain high communication standards (especially coverage), the issue of high power consumption remains unresolved [4]. The radio is one of the major power-consuming sections of the sensor in WSNs that can be handled using energy-efficient medium access control (MAC) protocols. Several MAC protocols, introduced to reduce the energy consumption, improve the lifetime of WSNs [5]. Unfortunately, most of the application-dependent [6] MAC protocols for WSNs are not energy efficient and thus do not effectively improve the lifetime of WSNs. The protocols should be scalable to adjust to changes in the network, such as the insertion of new nodes and the deletion of existing nodes [7,8]. The reduction in energy achieved by the MAC protocols increases the latency, particularly in multi-hop data communication [9]. These design constraints must be considered when developing new MAC protocols.

MAC protocols are classified into different categories, such as schedule-based, contention-based, mobility-aware, and hybrid protocols [10,11], however, many of the contention-based MAC protocols are based on sensor-MAC (S-MAC), which are designed for specific WSN applications [12]. Contention-based protocols have free access to acquire the medium [13]. The nodes, which follow contention-based mechanisms, are not required to follow the cluster. These protocols are network adaptable to allow for the insertion and removal of sensor nodes from the network. However, in contention-based MAC protocols, when nodes are available on channel but do not know the activities (schedule) of each other, nodes do not know when to turn on/off the radio, thus increasing the energy consumption. Schedule-based MAC protocols are more suitable for reducing idle listening [14]. However, in such protocols, node problems occur due to the presence of a tight schedule; once a node misses its schedule, then it must wait for the next turn, thus increasing the energy consumption. Additionally, schedule-based MAC protocols are not adaptable due to changes in network topology [15].

Hybrid protocols leverage the characteristics of time division multiple access (TDMA) and carrier sense multiple access (CSMA) [16]. Existing hybrid MAC protocols are based on the clustering approach [17,18], where time is divided into different time slots for each node in the cluster. Each node is responsible for using its own allotted time slot. Clustering reduces the idle listening and collisions. The transceiver also receives the sleep schedule without any additional overhead. However, such a mechanism experiences several drawbacks, as discussed in [16]. First, it is critical to determine an effective time schedule in a scalable manner. A centralized node is often needed to determine a collision-free schedule. It is extremely difficult to create an effective schedule with channel reuse or a high degree of concurrency (the ideal solution is NP-hard) [19]. Second, TDMA requires clock synchronization, which is an important feature of several sensor applications. However, tight synchronization results in energy overhead because it necessitates recurring message exchanges. Third, issues may arise due to frequent topology changes resulting from time-fluctuating channel conditions, such as battery outages, changes in the physical environment, and node failure. Controlling dynamic topology changes is costly and may even require a global change. Fourth, it is difficult to determine the intercession relation among neighboring nodes due to different communication and radio interference ranges from each other and other interfering nodes that may not be involved with direct communication (this situation is known as interference anomaly) [20]. Fifth, during low contention, TDMA results in lower channel utilization and increased delays. These problems with TDMA demonstrate that TDMA is not a reasonable choice when used individually, even if an efficient TDMA schedule is used. CSMA is attractive due to its flexibility, simplicity, and robustness. CSMA does not need considerable setup support, such as clock synchronization and global topology information. The dynamic joining and leaving of nodes is handled efficiently without additional operations. However, these benefits may come at the cost of an increased amount of trial and error; a trial may face collisions when more than two nodes attempt to access the channel simultaneously, causing signal fidelity to decay at the destination. Collisions can occur in any two-hop neighboring nodes. Although collisions at a one-hop neighbor node can easily be reduced by using carrier sensing before transmission, carrier sensing is not controlled beyond one hop. This issue, called the hidden terminal problem, affects throughput, particularly in high-data-rate sensor applications. RTS/CTS is an additional method to deploy with *virtual carrier sensing* in (CSMA/CA). The RTS frame consists of five fields include frame control, receiver address, duration, FCS and transmitter address. The CTS frame consists of four fields include frame control receiver address, FCS and duration. Although RTS/CTS can reduce the hidden terminal problem, it creates high overhead (40%–75%) in channel utilization due to control packets in WSNs [21,22].

Scalability and mobility are major issues whenever a node changes. Hybrid MAC protocols also experience inter-cluster communications and require tight time synchronization. These hybrid MAC protocols also use long preambles (signals used to synchronize transmission timing between two or more nodes and systems) that consume bandwidth and increase channel utilization [23]. To address these issues, the BN-MAC mobility-aware hybrid protocol introduces cross-layering support to control mobility and uses short preamble messages to reduce bandwidth consumption.

Combining CSMA and TDMA and including additional features, BN-MAC is a highly robust mobility-aware protocol for controlling timing failures, slot allocation failures, time-varying channel disorder, synchronization, and topological changes. In worst-case scenarios, the performance of BN-MAC will not be reduced because this protocol needs local synchronization at one-hop neighborhoods. Our analyses prove that the overall performance of BN-MAC will still be comparable to other hybrid MAC protocols when clocks are unsynchronized and slot allocation failure occurs.

The remainder of this paper is organized as follows: in Section 2, we discuss the goals, challenges, and contributions of this research. In Section 3, we present related work on hybrid MAC protocols. In Section 4, the system model is discussed. In Section 5, the BN-MAC protocol design is presented. In Section 6, the automatic active and sleep (AAS) model is presented. Section 7 presents the intelligent decision-making (IDM) model to automatically place nodes into either active or passive mode. Section 8 describes the simulation setup and analysis of the results. In Section 9, we discuss the results. Finally, our conclusions are presented in Section 10.

## 2. Research Goals, Challenges, and Contributions

One of the key goals of introducing BN-MAC is to support the multiple application domains of WSNs. We focus on several characteristics and factors that affect the performance of existing hybrid MAC protocols and BN-MAC. Factors that affect energy consumption and scalability include idle listening, overhearing, congestion, and mobility. The key challenge is determining how to integrate all of the proposed models to work as a single unit. Mobility is also difficult to address due to limitations and constraints at the MAC layer for maintaining scalability.

BN-MAC is proposed as a hybrid protocol involving a contention part and a scheduled part. The contention part is semi-synchronized [24] with a low duty cycle that helps to achieve faster access to the medium and manages the synchronization among nodes. The semi-synchronous feature is preferable for several application areas to reduce latency and energy consumption and maximize throughput. Second, the schedule part works with a dual message mechanism. Whenever the sensor node requires the schedule of its neighboring nodes, the sensor node uses the Anycast message mechanism because the sensor node can send a control message to only the nearest node in the group of potential receivers or may choose several nodes, depending on the situation. When the data are sent, the node uses the unicast message mechanism to forward the same data to all possible destinations. In addition, the neighbor discovery process consists of a short preamble message that consumes less energy. The dual mechanism avoids network congestion and increases the lifetime of WSNs. Third, BN-MAC discovers the presence and level of mobility of the sensor nodes within its neighbors using the received signal strength indicator (RSSI) and link quality indicator (LQI), both of which are obtained from the neighbor nodes at the time of synchronization.

BN-MAC performs localized reuse time slot allocation without changing the slots of the nodes that already exist if the node intends to perform further communication. This feature reduces latency and control messages and increases throughput. Fourth, new energy level information (ELI) algorithm is used for the dynamic selection of the coordinator, known as the boarder node (BN). BN dynamically works as a coordinator (head or leader) on a specific position. BN stays at the position as long as it uses its sources “energy” for performing some specific task for a definite period inside the network region then it vacates the position when the energy is reduced for the next node to become BN. In BN-MAC, the node with the highest energy level in its region will have a large probability of becoming the BN. BN-MAC approach can handle diverse situations more effectively. Additionally, three models are included in BN-MAC: AAS, LDSNS, and IDM. AAS is a simple yet efficient model for solving an idle listening problem. With the AAS model, sensor nodes are forced to go into the sleep state after performing the events that can prolong the lifetime of the network. This model significantly outperforms the previous sleep-wake up approaches designed for controlling the idle listening time. LDSNS is used to determine the shortest distance of the sensor node to one-hop neighbor nodes. The sensor node does not have the ability to send data over long distances; thus, LDSNS finds a close one-hop neighbor node to reduce energy consumption and improve the network lifetime.

The IDM model is used to sense the nature of the environment. This ability is critical because the sensor node is capable of obtaining energy from the Sun, which allows the sensor node to preserve its battery energy when automating the passive mode in an outdoor environment. The mode of the sensor node is typically set manually at the time of installation according to the nature of the environment; however, the IDM model automates the sensor node to reduce the energy consumption and expand the network lifetime.

## 3. Related Work

Although the deployment of WSNs has highly fascinated academia and industry, WSN platform has been experiencing several kinds of challenges due to many limitations and constraints. The WSN performance depends on an efficiency of the MAC protocol. The necessity of multi-featured MAC protocol is of paramount importance to handle mobility based scenarios for several real time WSN applications. The salient features of most related work are discussed. We emphasize some of the known hybrid MAC protocols. The hybrid protocol named Z-MAC is introduced that integrates the features of both TDMA and CSMA techniques [25]. In Z-MAC, CSMA is used as a baseline and TDMA resolves conflicts by scheduling the channel access. The protocol is based on the owner slot concept. Z-MAC uses novel flexible local time-frame synchronization without global synchronization. But, it requires the global clock synchronization. Z-MAC also introduces node highest priority scheme. If any node competes for accessing the channel, then the highest priority based node first gets the access to the channel. In a highly competitive environment, the node priority scheme decreases the network congestion. However, Z-MAC experiences latency issues due to the use of long preambles. Further, Z-MAC has another network adaptability problem because the nodes are tightly scheduled with each. As a result, Z-MAC decreases the throughput and increases excess energy consumption during the mobility.

Advertisement-based MAC (A-MAC) hybrid protocol is introduced in [26] for controlling collision, overhearing and marginally idle-listening issue. In A-MAC, TDMA is used as baseline while CSMA improves the channel access. Each node is assigned certain number of time slots within the two-hop destination. The assigned time slots are used to transmit the data without disturbing the other nodes. A-MAC also uses an advertisement message that helps the sender to inform the neighboring nodes regarding its transmission schedule. The major advantage of A-MAC protocol is to inform the nodes in advance in order to make receiver and sender ready for data transmission. This inclusion avoids the idle listening and overhearing. However, the overhead of control packets increases the latency and consumes extra energy. Further, A-MAC is only designed for monitoring the surveillance applications, but it does not have enough support for mobility and real time communication.

Speck MAC is a deviation of B-MAC protocol [27]. The Speck MAC aimed to reduce energy consumption and overhearing problem during heavy traffic. However, it consumes extra energy by sending wake-up frames [28] and also experiences excess latency. Speck MAC does not support for the real time and mobility based applications. ADC-SMAC [29] is an improved version of S-MAC that adds two new features to S-MAC. First, the node is capable to calculate its energy consumption and an average sleep time before sending synchronized packets. Second, the node adjusts the duty cycle based on network conditions then announces its schedule by sending broadcast messages to neighbor nodes. These two features reduce the energy consumption, but increase latency. Additionally, ADC-SMAC behaves poorly in mobile environments.

Low-power real-time medium access control (LPRT) protocol is proposed for actuation and wireless systems using star topology [30]. The LPRT-MAC introduces the super frame concept that uses mini slots for transmission to the base station. LPRT-MAC reduces the energy consumption when coordinating with the channel. Star topology avoids the network overhead. However, the LPRT-MAC performance is limited and not suitable for long multi-hop WSNs. Additionally, it is also not compatible with other communication topologies. Based on the literature survey of hybrid MAC protocols, we conclude that the reported hybrid MAC protocols are not good candidates for mobility and real time applications under congested and heavy traffic network load. To support several mobility and real time applications, we have introduced BN-MAC protocol that reduces energy consumption and improves scalability. BN-MAC also controls the congestion based on LDSNS and energy aware-routing protocol (EAP) [31] to maximize the throughput, reduce the latency and prolongs the network lifetime.

## 4. System Model for BN-MAC

We adopt an ad hoc-based network architecture that comprises sensor nodes with limited power resources and a BN with more dispensing capability and higher energy. The nodes are scattered to monitor the different events and activities. The WSN is divided into different regions, with each region controlled by a BN that coordinates within the given region and adjacent regions. Numerous economical BT node rev3 sensors are deployed over the battlefield area to provide a high level of coverage. The BT node rev3 is a self-directed prototyping platform based on a microcontroller, a Bluetooth radio, and ZigBee. The Bluetooth-enabled sensors cover short-distance communication among the troops deployed at the nearest positions, whereas ZigBee covers the long distances among troops. A small number of fixed coordinators obtain accurate positions of their troops as well as the enemy and their weapons. Each end sensor node is logically connected with a digital addressable lighting interface controller (DALIC). A DALIC consists of a controller and supports single or multiple lighting devices. The controller monitors and controls each light by using bi-directional data exchange. The DALI protocol broadcasts messages simultaneously to the address multiple devices to find their locations. The DALIC helps to monitor and locate the exact position of the enemy. To determine the exact location, the DALIC requires an active bat location (ABL) system that automatically determines the location of the objects.

We also assume that all of the sensor nodes use seismic modality, and each sensor senses different events during every sampling period using a seismic frequency spectrum. We have considered multiple issues when designing region-based WSNs for a military scenario. The first consideration is that we have identified the area of the war and a possible solution. The second consideration is focused on the deployment issues of the network, such as the location of the sensor nodes determined before deployment. In this manner, the degree of coverage and connectivity is secured. The nodes are randomly scattered in the disaster area. To save energy, the nodes typically use short-range and one-hop communication rather than long-range communication. We use a one-hop destination search to schedule and deliver data.

**Figure 1.**
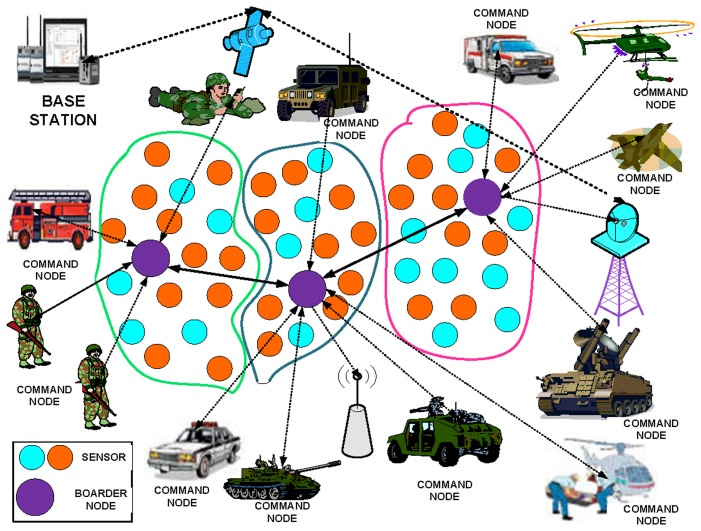
Proposed simulated WSN.

We have focused on a combined mobility and static scenario using the ns2 network simulator in the scenario depicted in Figure 1. Each static and moving object is connected with a command node. The command node is a heterogeneous node that obtains event information through homogenous nodes fixed in the field. Similarly, the command node forwards the collected information using the (homogenous) sensor nodes to the BN. In this scenario, the battlefield is dispersed into different regions. Each region covers several command nodes that gather information from the events. The message-forwarding process consists of intra- and intercommunication. Intra communication is used within the region, whereas intercommunication is used outside of the region. The mode of communication within the region is based on Anycast communication. Anycast is used to exploit the knowledge of immediate channel condition in choosing the appropriate downstream neighbor on smaller time scales. Additionally, the main notion behind MAC layer anycasting is to accomplish the objectives of network layer, while invoking short-term improvement at the MAC layer, based on the local channel settings. Anycast also provides the option of specifying multiple downstream destinations to the MAC protocol. Anycast allows for increased load balancing to minimize the work load and complexity of the network for reliable data transfer. Unlike Anycast, multicast increases latency. Thus, each node stores and forwards the packets to several nodes, resulting in increased energy consumption. This battlefield scenario requires mobility and scalability. The cross-layering support of BN-MAC successfully resolves this issue using the pheromone termite (PT) mobility model. The PT model provides robust and faster routing over WSNs. This model is specially designed to control the scalability of WSNs and the mobility of nodes. The PT analytical model monitors the behavior of the WSN using the packet generation rate and the pheromone sensitivity over single and multiple links [32]. The PT routing model monitors the different activities of the troops and maintains a faster recovery process using the packet generation rate and pheromone sensitivity. BN-MAC uses the AAS model to address idle listening in nodes, as discussed in Section 6. The AAS model lets the nodes go into the sleep state after monitoring and processing the collected information. This approach allows the nodes to reduce the amount of energy consumed in idle listening. In this scenario, some of the sensor nodes are deployed in the open battlefield area, whereas some are grounded or fixed to buildings to monitor different processes, as such situations demand the sensor nodes to act differently. BN-MAC uses the IDM model to sense the nature of the environment, which allows the mode of the sensor node to be automatically switched either into the active or passive mode. The IDM model also reduces WSNs' energy consumption.

## 5. BN-MAC Protocol Design

BN-MAC is proposed with the aim of supporting multiple applications, particularly military applications, which require mobility-aware and static devices to be controlled from remote places. MAC design in WSNs is ant involved process because WSNs are based on mechanisms that are entirely different from the traditional networks. WSNs have limitations due to storage, computational capability, and energy resources. Therefore, the MAC protocols should be well organized to distribute the bandwidth fairly and be energy efficient, with appealing features that may stimulate the robust design of the communication media. One of the key factors for introducing BN-MAC is to reduce energy consumption while addressing idle listening, overhearing, mobility, and congestion concerns. BN-MAC also shortens the latency while guaranteeing the reliability of the WSN.

BN-MAC improves the existing Z-MAC, A-MAC, Speck-MAC, ADC-SMAC, and LPRT-MAC protocols by adding new features. The mechanism of BN-MAC supports the hybrid topology that combines the features of TDMA and CSMA. The network is constructed as a flat single-hop topology. The features of TDMA are used to improve the contention, whereas CSMA works as a baseline. BN-MAC follows the concept of the owner slot. The node has complete access to its owner slot, similar to TDMA-based approaches. The remaining slots are accessed through the CSMA approach. The CSMA approach preserves energy and controls collisions. In addition, BN-MAC eliminates idle listening in each region to achieve a considerable energy saving. Bi-directional traffic inside each region of the WSN promotes smooth data exchange and efficient use of the bandwidth. Additionally, BN-MAC uses dynamic contention free slot exchange, which increases network scalability under even a heavy traffic load.

BN-MAC consists of the following phases: finding the list of one-hop neighbors, intra-semi-synchronous transmission scheduling, inter-synchronous transmission scheduling, and selection of a BN. These operations are performed once during the setup process and are not performed again until the network topology is physically changed. In this approach, the initial costs for running these operations are balanced while achieving a better throughput and reduced energy consumption during intra- and inter-transmission.

### 5.1. Finding the List of One Hop-Neighbors

When a node intends to start communication with its neighbor node after accessing the channel, the node sends an Anycast message to its one-hop neighbor nodes to obtain the details of neighboring nodes. This process helps to reduce overhead and manage network load balancing. The process of sending the Anycast ensures that the intended neighboring nodes are able to talk with each other, even if they possess different sleeping and communication schedules. The neighbor discovery process consists of short messages (short preambles), which consume less network bandwidth and improve the throughput.

**Figure 2.**
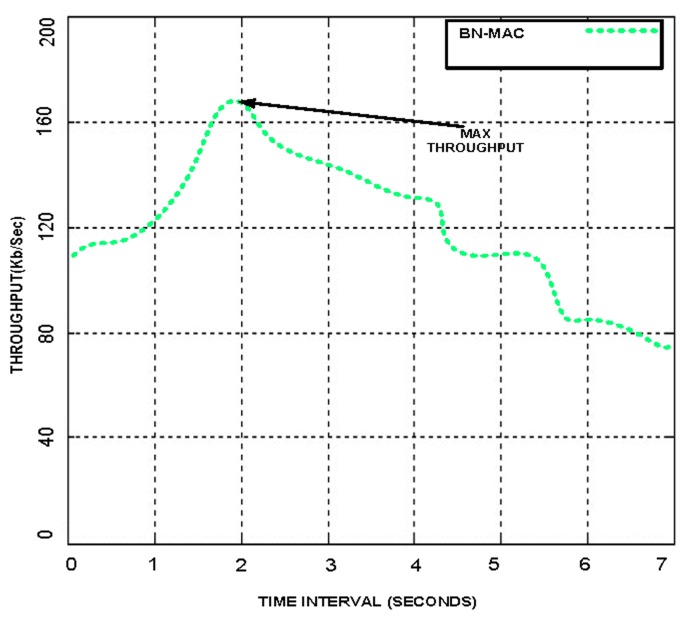
Throughput at different time intervals.

**Figure 3.**
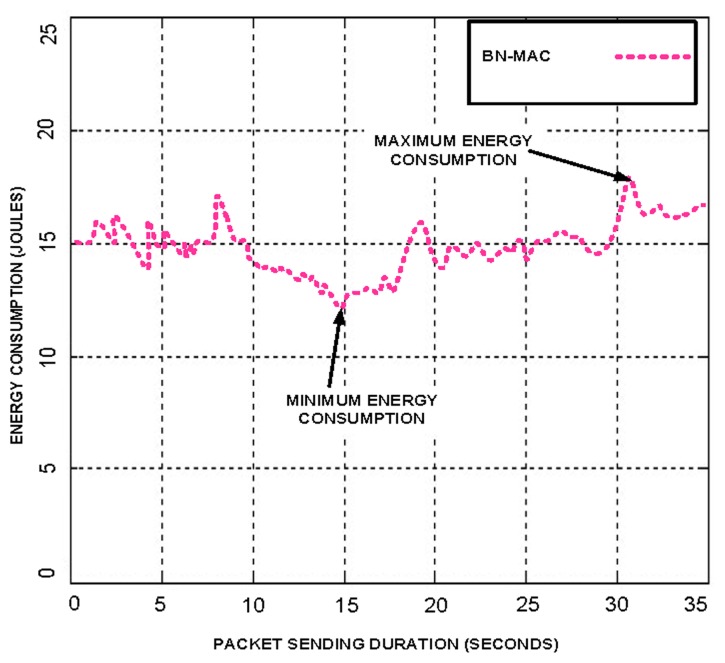
Packet sending duration *versus* energy consumption.

Each node randomly sends a short preamble for finding the list of intended neighbor nodes using Anycast after two seconds for 15 s. This timing is used obtain maximum throughput; packet sending intervals from 1 to 10 s were considered, but the time interval of 2 s provides the maximum throughput. We have also set the packet sending time at 15 s to facilitate the successful completion of the packet sending process. If we set the time less than or higher than 15 s, then the node energy is wasted. The node is unable to complete the packet sending cycle when the time is less than 15 s, and when the time is greater than 15 s, the node comes into the idle situation because after finishing the packet sending task and thus waits on the channel until the level of set time is reached. We present the performance of the BN-MAC at different time intervals and packet sending durations in Figures 2 and 3. A comparison of BN-MAC and Z-MAC, the nodes of which use 30 s for the neighbor discovery process, indicates that Z-MAC has higher energy consumption.

The node discovery process in BN-MAC consists of a one-hop neighbor node, but nodes are able to obtain two-hop neighbor information that is helpful for expanding cross-layering support. The two-hop information that has been obtained is also used for slot allocation, which enables the node to increase mobility because the node retains the information even when the two-hop node is moving. BN-MAC is scalable because the one-hop topological change is easy to handle; each node knows the schedule of the one-hop neighbor node. BN-MAC uses a promising time scheduler because the assigned slot does not exceed the one-hop neighborhood. BN-MAC also performs the without changing the time slots of existing nodes. The localized time slot allocation which is used with channels to synchronize for the whole network. Otherwise, conflict between different traffic flows can occur. It also helps the node to gather allocation information for all 1-hop and 2-hop neighbor nodes. This feature of slot allocation re-use improves throughput and reduces node latency.

### 5.2. Intra-Semi-Synchronized Transmission Schedule

This mode is based on a semi-synchronized low duty cycle (the ratio between active time and the complete active/sleeping time; a low duty-cycle MAC protocols obviously has a much extended lifetime for operation, but pauses for the all-node-active assumption). The intra-semi-synchronized process starts with channel sampling. The node wakes up for a short period of time to sample the medium. Channel sampling is performed once during the channel allocation time. After channel sampling, each node initially sends a short preamble message asynchronously using the Anycast approach within the one-hop neighbor node to obtain the list of one-hop neighbor nodes. When the sender receives a reply from the one-hop neighbor nodes, the sender attempts to fix the schedule with the intended one-hop neighbor nodes (nodes that are chosen for future communication) before sending the data. Each node knows the wake-up and sleep schedule of its intended neighbors. These dual features of sending a short preamble asynchronously to obtain the list of neighbor nodes and fixing the schedule synchronously reduce the network overhead. When the sender completes the scheduling process with the intended nodes, the sender chooses the shortest efficient path for sending the data using the LDSNS model, as explained in [33]. This model helps to reduce energy consumption and the links with the network layer. The use of a short preamble message allows for reductions in overhead and latency at each hop. The short-preamble-enabled MAC protocols have an advantage over the long-preamble-enabled MAC protocols due to their low-power duty cycle mechanism. The existing lower power listening (LPL) technique uses a long preamble and suffers from the overhearing problem, which increases energy consumption in non-targeted receivers, such as Z-MAC. LPL also increases latency at each hop [34]. In the long-preamble techniques, the node must wait until the long preamble is received before it starts receiving data and acknowledgments. This approach increases energy consumption on both the sender and receiver sides. Targeted receivers are also affected because the targeted receivers have to wait until the long preamble is received, causing increased energy consumption.

X-MAC uses a short preamble message to reduce the energy consumption and latency, but one disadvantage of X-MAC is that the destination address of the node is inserted into each short preamble message. X-MAC forces all nodes to check the preamble to determine whether they are targeted nodes, which increases energy consumption and the duty cycle (wake-up process). X-MAC is based on an asynchronous mechanism, and no schedule of neighbor nodes is maintained, making it more difficult for each node to send data without prior scheduling information. Unlike X-MAC, BN-MAC deploys both asynchronous features for sending short preamble messages to obtain the list of one-hop neighbor nodes and synchronous features for fixing the schedule with the intended neighbors.

The MAC protocol should be capable of handling spatial correlation while also adjusting to changes in the number of competing nodes [35]. When multiple nodes want to communicate with the same neighbor node within the region, BN-MAC uses a slotted contention window. Then, the nodes randomly select a slot in the contention window.

**Figure 4.**
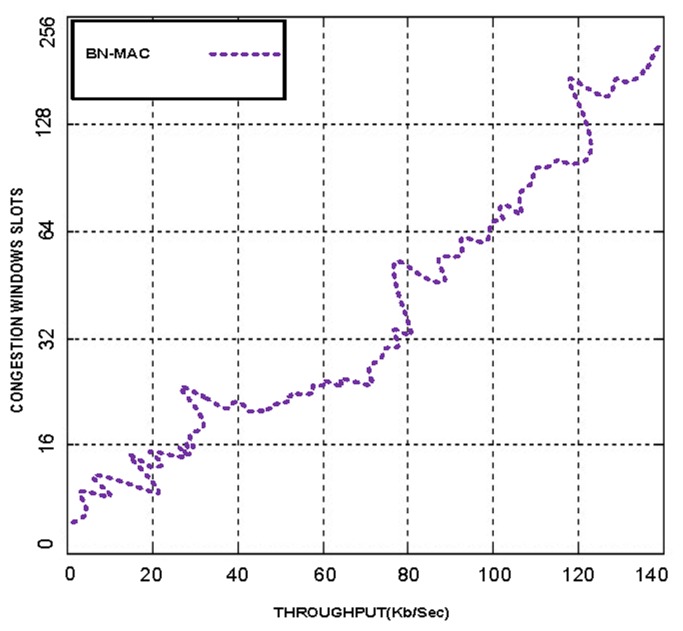
Throughput at different congestion window slots.

The winner of the slot obtains access to the medium for communication. Thus, there is small probability of collision at the medium. BN-MAC has more contention slots to compete, which reduces congestion in the WSNs. BN-MAC has another feature the helps to reduce packet loss. If multiple nodes attempt to select the same slot, BN-MAC uses sampling and randomization such that each node has an equal probability of accessing the channel. Furthermore, BN-MAC uses 256 congestion window slots, whereas the other MAC protocols use 1–32 contention windows for randomized listening before sending the preamble messages. This increased number of slots reduces congestion and latency and allows higher throughput to be obtained. We have used different numbers of congestion window slots, with 30% of the active sensor node contenders allocated to each window slot. These experiments indicated that BN-MAC produces the maximum throughput when 256 slots are used, as shown in Figure 4. Similarly, we have checked the performance of hybrid MAC protocols on their existing window size slots and compared the hybrid MAC protocols with BN-MAC.

**Figure 5.**
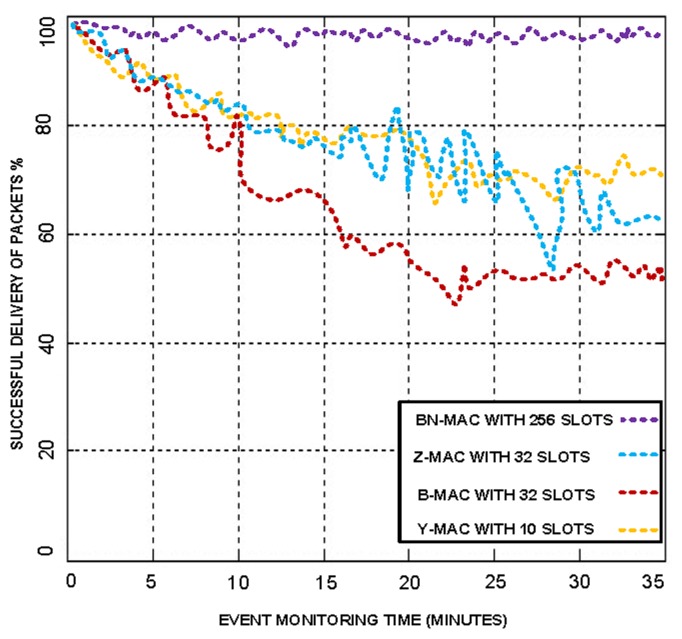
Successful delivery of packets *versus* event monitoring time.

The simulation results demonstrated that BN-MAC successfully delivers 99.8% of packets, whereas other MAC protocols only successfully deliver 46%–72.7% of packets, as shown in Figure 5. Hence, the use of 256 window slots increases the throughput considerably.

**Figure 6.**
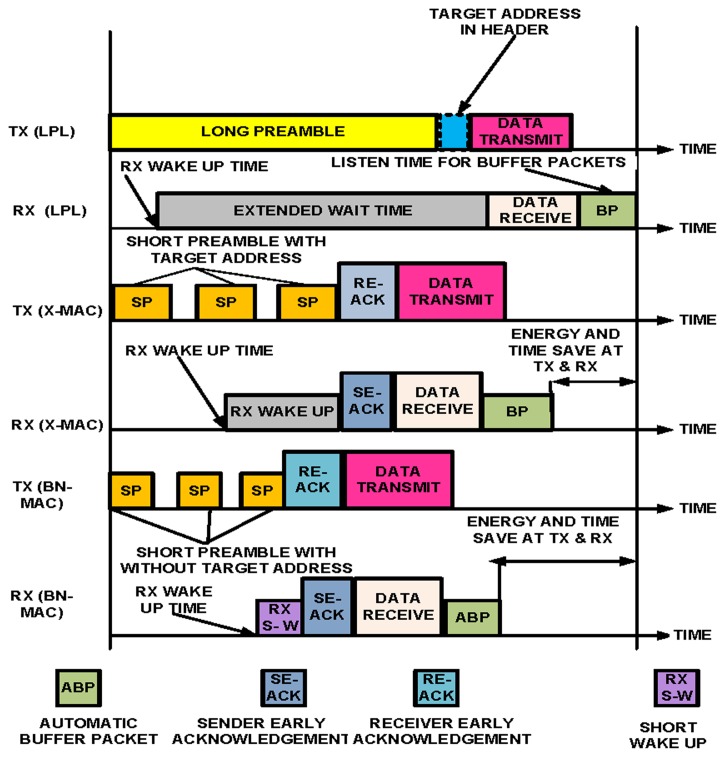
Comparison of the timelines of duty-cycle MAC protocols.

Sensor nodes also perform automatic buffering within the region during intra-communication to reduce the drop rate and prolong the network lifetime. We demonstrate the process of long permeable (LPL), short permeable (X-MAC), and BN-MAC in Figure 6.

X-MAC uses a short preamble with a target address to access the channel to communicate with another node. However, all of the nodes en route will remain awake until the short preambles are received by the destination node, which results in increased energy consumption. X-MAC also has a delay of transmission for sending the packets until the receiver wakes up [36]. The BN-MAC protocol does not use the target address of the node when sending a short preamble message. Thus, all of the nodes do not continue to wait; instead, only the intended node wakes up to receive the short preamble. Thus, each node is in sleep mode for a longer period of time. In addition, BN-MAC uses an automatic packet buffering process similar to that used in [37]; this process reduces the wake-up time and increases the network lifetime. In the automatic buffering process, the node uses a promiscuous mode that enables the node to listen to all ongoing data traffic and coordinates, if requested. Furthermore, the node saves a copy of the received packet regardless of the intended destination of the data packet until receipt of the packet is acknowledged by the destination node. Such buffering requires a relay that is used by the saturated conditions because each node is able to cooperate in sending data packets to other buffers. As mentioned above, a short preamble consumes less energy and prolongs the network lifetime. Let us find the energy consumed for channel sampling and short preamble messages.

The consumed energy for channel sampling is “*Ψ*”, the check period is É, and the average energy consumed for channel sampling is “*γ*”:

(1) $$\gamma =\frac{\psi }{\overset{´}{E}}$$

The energy consumed for a short preamble “*τ*” consists of the average energy consumed for channel sampling, “*γ*”, and the energy consumed in sending and receiving synchronization, *“ω*”:

(2) $$\tau =\gamma *2\omega *\Delta_{t}^{2}$$

We use clock drift, “ $\Delta_{t}^{2}$”, which is the time consumed sending and receiving the synchronization, and “2ω” is the energy consumed by the sender and receiver for synchronization.

During intra-communication, the node that transmits its clock to the one-hop neighbor is called the parent, and the receiving node at the one-hop neighborhood is called the child. The nodes that are synchronized with the clock often use a short preamble without the target address of the node that reduces the energy consumption.

Let us assume that the average energy consumed by the parent and child nodes for one work cycle is “*α*” and “*β*”, respectively. The average short preamble reception time could be reduced because the receiving node wakes up based on the stored schedule of the neighbor nodes. The average energy consumed by the parent and child nodes can be obtained as follows:

(3) $$\alpha =\sum_{k=0}^{n}p_{k}\frac{\left(\nabla \sigma \cdot \mu *\delta \nu^{2})*(\gamma *2\omega *\Delta_{t}^{2}\right)}{\Delta_{t}}$$

(4) $$\alpha =\sum_{k=0}^{n}p_{k}\frac{\left(\nabla \sigma \cdot \mu *\delta \nu^{2})*(\gamma *2\omega *\Delta_{t}^{2})+(\gamma *\omega *\Delta_{t}\right)}{\Delta_{t}}$$

where “*k*” is the starting point of the short preamble, “*n*” is the ending point of the short preamble, “p_k_” is the short preamble, “*∇σ*” is the size of the preamble, “*μ*” is the nature of the environment, “*δν*^2^” is the speed of the short preamble, and “Δ_t_” is the total time spent sending the short preamble.

From Equations (3) and (4), we can obtain the total energy consumed sending the short preamble during the event monitoring time. Equation (3) represents the energy consumed by the parent node in sending the short preamble within the one-hop neighbor nodes, whereas Equation (4) represents the energy consumed by the child node in receiving and sending by the short preamble to the two-hop neighbor nodes and also acknowledges the parent node.

**Figure 7.**
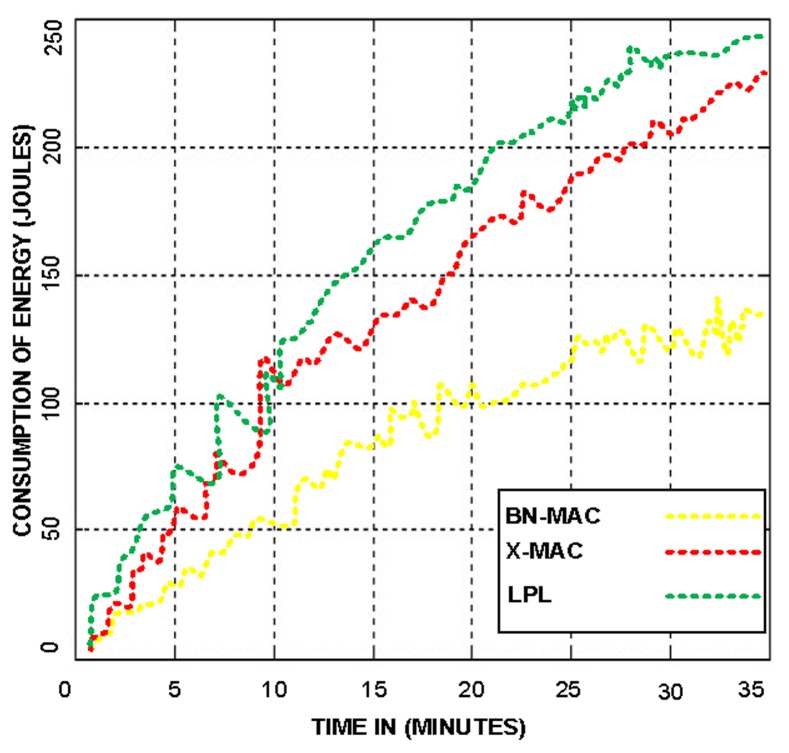
Energy consumption for BN-MAC and low-duty-cycle MAC protocols.

**Figure 8.**
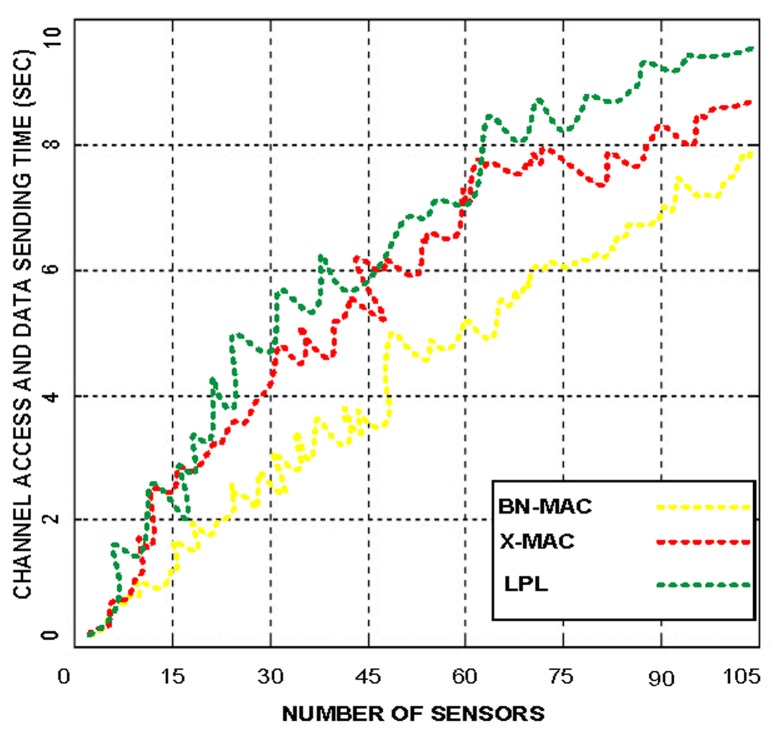
Channel accessing and data delivery time for BN-MAC and other low-duty-cycle MAC protocols.

BN-MAC can clearly identify the consumed energy of the short preamble prior to sending the data. BN-MAC has an advantage over low-duty-cycle long-preamble-enabled MAC protocols and X-MAC. The reduced energy consumption and time requirements of BN-MAC compared to the other protocols is shown in Figure 7. Figure 8 presents the superiority of BN-MAC compared with other low-duty-cycle MAC protocols in terms of time consumed in sending the short preamble to confirm the synchronization process for forwarding the data.

All of the nodes in BN-MAC maintain the same time frame during synchronization and maintain a time slot of 0. Each node maintains its own local frame, which matches the frame size of the neighborhood to avoid potential conflicts while contending with neighbors.

The nodes compete for CSMA equally during the contention phase because the random exponential back-off (an algorithm that uses response to multiplicatively reduce the node's frequent access to the channel dynamically in order to find the acceptable node to access the channel, as the part of network congestion avoidance) preserves the right of each node to compete fairly for scheduled slots. Intra-semi-synchronous communication is performed inside the region because BN-MAC is designed purely for the region-based network, as many WSN application areas require a region-based network. The intra-semi-synchronized transmission schedule is compatible with all types of radios, such as CC2420 and CC2500.

### 5.3. Inter-Synchronized Transmission Schedule

BN-MAC is used with WSNs that consist of different regions. The previous section highlights how to access the channel and forward the data inside regions. This section explains how to set the schedules within and outside regions. Each region of the WSN includes a BN. Inter-synchronized transmission is performed from one region to other regions. The BN receives intraregional data packets within the region, and the BN forwards the inter data packets outside of the region.

**Figure 9.**
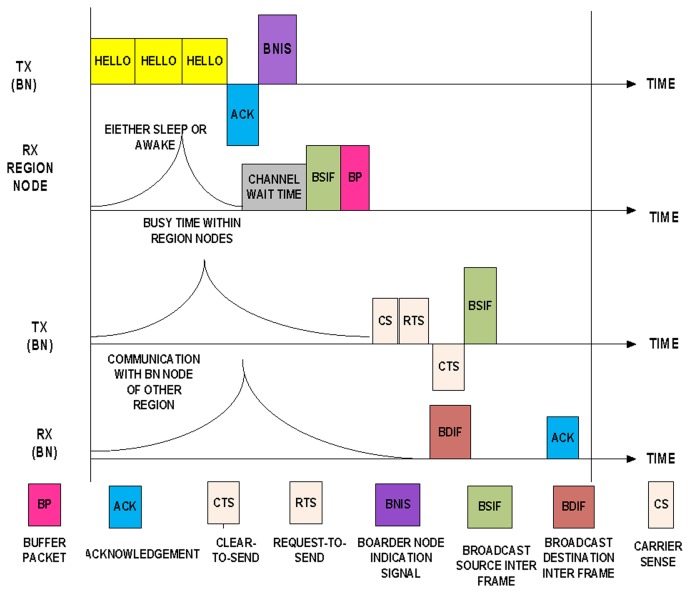
Inter synchronized transmission schedule with the region node and BNs.

When communicating within the region, the BN first broadcasts three “hello” messages to warn the nearest region nodes to prepare for receiving the BN indication signal (BNIS). The BN does not wait to receive an acknowledgment from all of the region nodes. If the BN receives a single acknowledgment from one of the nearest nodes, it assumes that the “hello” message has been delivered successfully. Thus, if any node is unable to obtain the “hello” message, the neighbor node informs other nodes of the schedule exchange time. In this manner, each node knows the BNIS. The BNIS consists of the current time, the next distribution time, the next collection time, and the schedule for obtaining intraregional data packets from the nodes of the region, as shown in Figure 9.

The BNIS also has the responsibility of exchanging traffic slots between the source and the destination and describing the related offset time. Once the BN announces its schedule for the nodes of the region, all of the nodes are responsible for following the given schedule. At the end of the scheduled time of the region nodes, the BN synchronizes with another BN of a region to exchange an interregional synchronous schedule to send and receive data communication. After the contention period starts, the node responsible for the data exchange requests the schedule-slot for the next scheduled distribution time.

The nodes only remain active during the BNIS. When the BN intends to communicate with another BN of a region, the BN begins the interregional synchronized transmission schedule by using carrier sensing. The BN forwards the message of request-to-send (RTS). In response, the BN receives a clear-to-send (CTS) message from the BN of the other region. There is no hidden terminal problem in BN-MAC because the BNs of all regions broadcast the messages to provide each BN with the schedule of every region. Through this process, all of the BNs know the other BNISs. After receiving the CTS, the transmitter of the BN forwards the broadcast source inter frame (BSIF) to another region. (BSIF is the frame used by BN to synchronize for sending the data to another BN of adjacent region). The receiver BN receives the broadcast destination inter frame (BDIF) during the interval with the CTS and RTS and acknowledges the received packets (BDIF is the frame received by BN after sending BSIF frame, it means BN is allowed to send the data to another BN of adjacent region).

**Figure 10.**
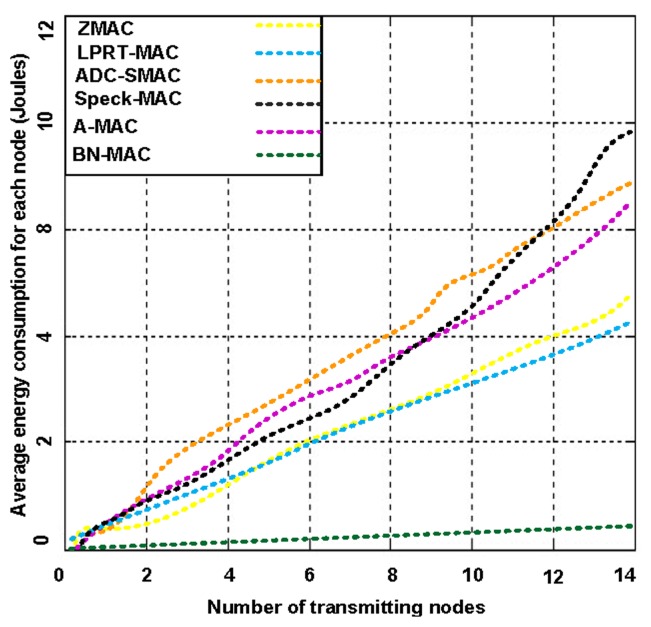
Energy consumption during heavy traffic using a low duty cycle.

We tested the intercommunication performance of BN-MAC and other hybrid protocols in terms of throughput and average energy consumption. We use varying numbers of transmitting nodes at a low duty cycle. Figure 10 presents the average energy consumption for each transmitter node, illustrating that BN-MAC is superior to the other hybrid MAC protocols at a low duty cycle.

**Figure 11.**
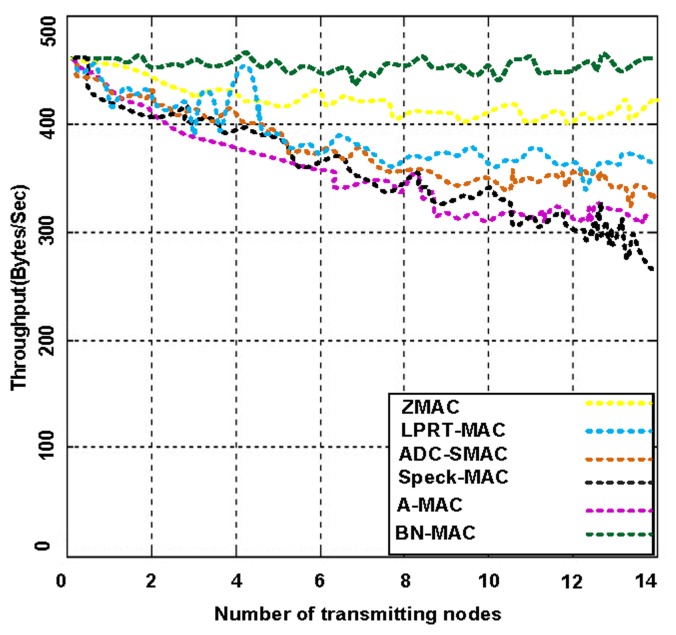
Throughput under heavy traffic using a low duty cycle.

As mentioned above, BN-MAC has an intra-semi-synchronous transmission schedule that follows the low-duty-cycle mechanism as well as the inter-synchronized transmission schedule that supports the low duty cycle under heavy traffic. BN-MAC is also outperforms the Z-MAC, A-MAC, Speck-MAC, ADC-SMAC, LPRT-MAC protocols at a low duty cycle under heavy traffic. BN-MAC also consumes less energy over a heavy traffic load using the low duty cycle. Figure 11 presents the potential increase in throughput obtained via BN-MAC during heavy traffic at a low duty cycle.

As the number of transmitting nodes increases, the energy spent for each node increases in competing hybrid MAC protocols compared with BN-MAC. Other protocols consume 18%–45% more energy than BN-MAC during heavy traffic, mainly because these protocols use many continuous preamble messages, whereas BN-MAC uses a short preamble to guarantee the efficient delivery of data. Another reason for BN-MAC's superior throughput performance is the use of BNs, which have automatic buffering capacity to store packets instead of discarding them.

### 5.4. Selection of the BN

The BN is selected periodically based on the BN volunteer selection process (BNVSP), which is similar to the process used in [38]. The BNVSP chooses the BN based on the available energy and memory allocation resources. No one node is compelled to declare itself as a BN based on a probability-based calculation. Each sensor node possesses a different energy level in the region after monitoring the event at any given time. The node energy level involves several factors, such as the sleep/wake-up schedule, amount of data received, and amount of data transmitted. The sensor nodes are actively involved in monitoring the events and forwarding the data of the targeted events to the BN. This situation leads to the death of the BN before the other nodes that are not actively involved. Thus, the BN faces a shortage of energy. To overcome this problem, the proposed BNVSP helps to determine the energy level of each node to select a BN based on the maximum residual energy of the sensor node in each region. Each sensor node announces its residual energy after completing the event monitoring process. This maximum residual energy amount determines whether the node should be considered a BN candidate or not but depends on the residual energy of the sensor node and the distance from the node to the base station.

The BN is selected based on the energy level using the BNVSP and level of energy information (LEI) algorithm, as shown in Table 1. The LEI function is used to determine the level of energy for each node, and the BNVSP is used to select the BN. We categorize the energy of sensors into six levels is given in Table 1. Algorithm 1 determines energy level for each sensor node.



**Algorithm 1:** Detection process of energy level for selection of boarder node.
1.Set N nodes = Number of nodes2.Computer VL= Voltage level3.If (VL >= 3.3 && VL <= 3.7) then Set VL = EL Declare EL = Very high4.if (VL >= 3.0 && VL <= 3.3 then Set VL = EL Declare EL = High5.if (VL >= 2.7 && VL <= 3.0) then Set VL = EL Declare EL = High moderate6.if (VL >= 2.4 && VL <= 2.7) then Set VL = EL Declare EL = Moderate7.if (VL >= 2.1 && VL <= 2.4) then Set VL = EL Declare EL = Low8.else (VL <= 2.0) Set VL = EL Declare EL = Lowest9.end if


When the energy level of the BN that is already working decreases, the responsibility is shifted from one BN to another BN using the election flag bit (EFB), a signal alert sent by BN in the network for the election of new BN when decreasing its energy level. The EFB specifies the process of the immediate BN election. The proactive method is used to select the next BN to reduce the overhead associated with this process. The base station broadcasts a short preamble message to each WSN node. Each node calculates its distance from the base station based on the signal strength. The node that receives a short preamble becomes a candidate for the BN. Other factors are also considered when selecting the final BN, including the energy of the BN consumed during the contention time for election (comparison time of the energy level), the energy consumption of the sensor node in each state, and the time spent in each state and the transmitted data at each step. All candidate BNs check their radio range and residual energy. The radio range is selected by the short preamble sent by the base station, and the residual energy is selected using the BNVSP, as shown in Table 1. The residual energy level for choosing the BNs is determined as follows:

(5) $$\text{LEI}=\sum_{k=1}^{3}E_{k}\;\pi_{k}$$

where *E_k_* is the energy consumed by each sensor node in each state and *π_k_* is the time spent in each state. Thus, 1 is “radio on” for receiving traffic, 2 is “radio off”, and 3 is transmitting the data packets.

Let us determine the residual energy level of each sensor node. “*L_e_*” is the residual energy level of each sensor node after performing the event, so we apply the following derivation:

(6) $$S_{e}=-\frac{1}{2}V\frac{z^{2}}{h_{n}}$$

where *S_e_* is the current energy level of the sensor, V is the voltage level of the sensor node before the event, *z*^2^ is the number of contention window slots, which is equal to, 2^8^ and *d*_1_ is the number of hops required to travel the data, and *h_n_* = *n*^2^ * *d*_1_ where *d*_1_ is the distance between each hop of the WSN. If the sensor node completes the event, then the sensor node decreases its energy level. Therefore, the energy used in the event is equal to the difference between the sensor node's final and initial energy levels:

(7) $$R_{e}=S_{e1}-S_{e2}=\frac{1}{2}V\frac{z^{e}}{d_{1}}\left[\frac{1}{{e_{1}}^{2}}-\frac{1}{{e_{2}}^{2}}\right]$$

where *R_e_* is the remaining sensor energy after the event, *S_e_*_1_ is the initial energy of the sensor, *S_e_*_2_ is the final energy of the sensor, and *e*_1_ and *e*_2_ are the initial and final energy levels, respectively.

If part or all of the consumed energy in the sensor node is renewable *Rn_e_*, then the new energy of the sensor node $\frac{C}{Rn_{e}}$ can be found as follows:

(8) $$\frac{1}{Rn_{e}}=\frac{1}{2}V\frac{z^{e}}{cd_{1}}\left[\frac{1}{{e_{1}}^{2}}-\frac{1}{{e_{2}}^{2}}\right]$$

where:

(9) $$\left[\frac{1}{{e_{1}}^{2}}-\frac{1}{{e_{2}}^{2}}\right]=L_{e}$$

We obtain the energy of the sensor node by substituting for $\frac{1}{2}V\frac{z^{e}}{cd_{1}}$ as follows:

(10) $$\frac{1}{Rn_{e}}=L_{e}(\frac{1}{2}V\frac{z^{e}}{cd_{1}})$$

Therefore, the level of energy can be expressed as:

(11) $$L_{e}=(\frac{Vz^{e}}{2cd_{1}})$$

**Figure 12.**
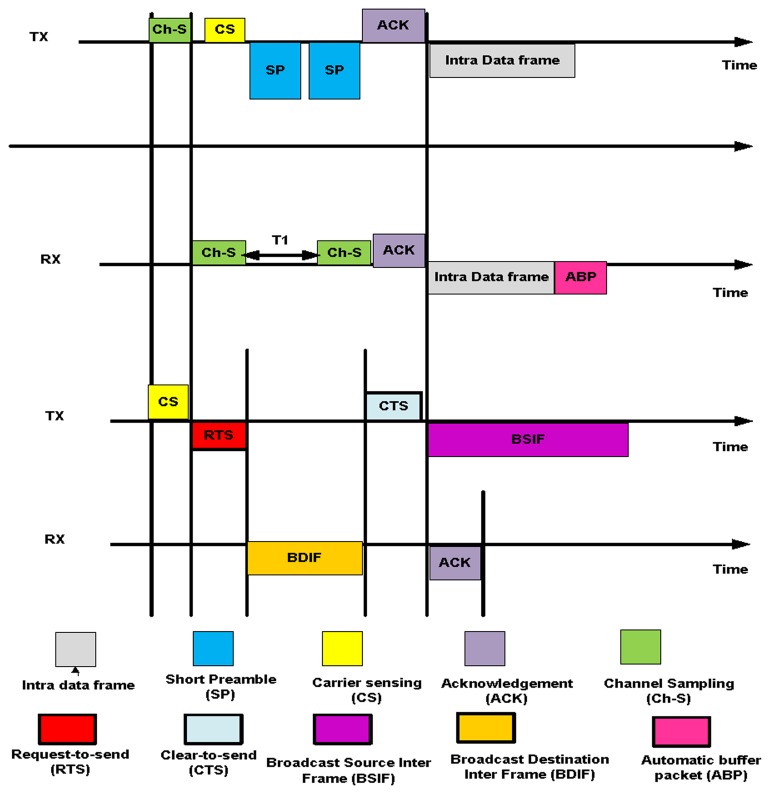
Message mechanism of the hybrid BN-MAC protocol.

The current BN also sends a signal for a new election when the battery is running down. After the election process, the new BN resumes its duty and the current BN terminates its function. In case of BN failure, the remaining nodes wait for four consecutive BNISs, and the BN is subsequently considered a malfunction. The new BN is automatically selected, allowing network disturbances to be avoided. We illustrate the complete mechanism of BN-MAC in Figure 12.

## 6. Automatic Active and Sleep (AAS) Model

The node is periodically set into the sleep state in the duty cycling protocol [39]. The node can maintain a tradeoff between data latency and energy consumption by fixing the state of either sleep or wake-up automatically. The node consumes less energy at a higher latency for data delivery with a lower duty cycle. Once a node wakes up during its active duty cycle time, it should listen to the channel for a specific period of time to determine whether other nodes are available for communication. This situation creates difficulties and increases the overhead of the MAC protocol due to idle listening, which is a major source of energy consumption. The nodes continue to monitor the channel for incoming traffic, which increases energy consumption. Some of the WSN applications require transfer at a low data rate, but the sensor nodes remain idle for a longer period of time after performing their specific events. It is not advisable to keep the sensor nodes in the idle state for a significant period of time. Thus, the AAS model is integrated with BN-MAC to shorten the unnecessary idle listening time. Sensor nodes normally operate in two modes: ON and OFF. If the transceiver of the sensor node is active “ON” without performing any events, time delay and wasted energy result [40]. The unnecessary waste of energy can be reduced if the transceiver of the sensor nodes is controlled using the “OFF” state. We use the AAS model by setting the threshold value for the “idle” and “OFF” states to save energy.

Let us assume that the energy consumed in the idle time of the sensor nodes should always be less than or equal to the “OFF time”. Then *E_itime_* ≤ *E_otime_*, where *E_itime_* is the consumption of the energy during the idle time and *E_itime_* is the energy consumed during the OFF time.

Let us assume that E_midle_(t) is the minimum energy required for the sensor nodes to remain in the idle state, E_idle/on_ is the energy consumed by switching from the idle state to the “ON” state, and CS_idle/on_ is the energy consumed by switching from the idle state to the “OFF” state. Thus, the energy consumed in idle time can be computed using Equation (12):

(12) $$E_{\text{itime}}*={\text{E}}_{\text{midle}}(\text{t})+{\text{E}}_{\text{idle}/\text{on}}*{\text{CS}}_{\text{idle}/\text{off}}$$

Let us assume that CS_on/off_ is the minimum amount of time required for the sensor nodes to consume energy by going from the “ON” state to the “OFF” state, CS_on/off_ is the time required for the sensor nodes to consume energy by going from the “OFF” state to the “ON” state, and *E_off_* is the energy saved by the nodes when they are in the sleep state. Thus, the total “OFF” time energy can be calculated using Equation (13):

(13) $$E_{\text{otime}}={\text{CS}}_{\text{on}/\text{off}}+{\text{CS}}_{\text{on}/\text{off}}+E_{\text{off}}$$

Let us assume that *E_otime_*, the total energy consumed during the “OFF” state, is larger than the energy consumed in the idle state, which was already proven and is given in Equation (14):

(14) $$E_{\text{otime}}\geq ({\text{CS}}_{\text{on}/\text{off}}+{\text{CS}}_{\text{off}/\text{on}}+E_{\text{off}})$$

Our goal is to transition the sensor nodes into the sleep state if no event is underway. Equations (12) and (13) indicate that the states of operations in the sensor nodes can be established automatically.

Let us set the transition states *α*(*alpha*) and *β*(*beta*) for sleep (OFF) and active (ON), respectively. An automatic change of transitions can be justified if Equation (15) is satisfied:

(15) $$CS_{a}\geq \max[0,(E_{\text{atime}}+E_{\text{itime}}-E_{\text{otime}})*CS_{\beta \alpha }]$$

where *E_atime_* is the energy consumed during the active time and *CS_βα_* is the negligible amount of energy consumed by going from the active (OFF) state to the sleep (ON) state. Thus, *CS_a_* is greater than or equal to the amount of energy consumed in the active and idle states minus the energy consumed during the total “OFF” time.

**Figure 13.**
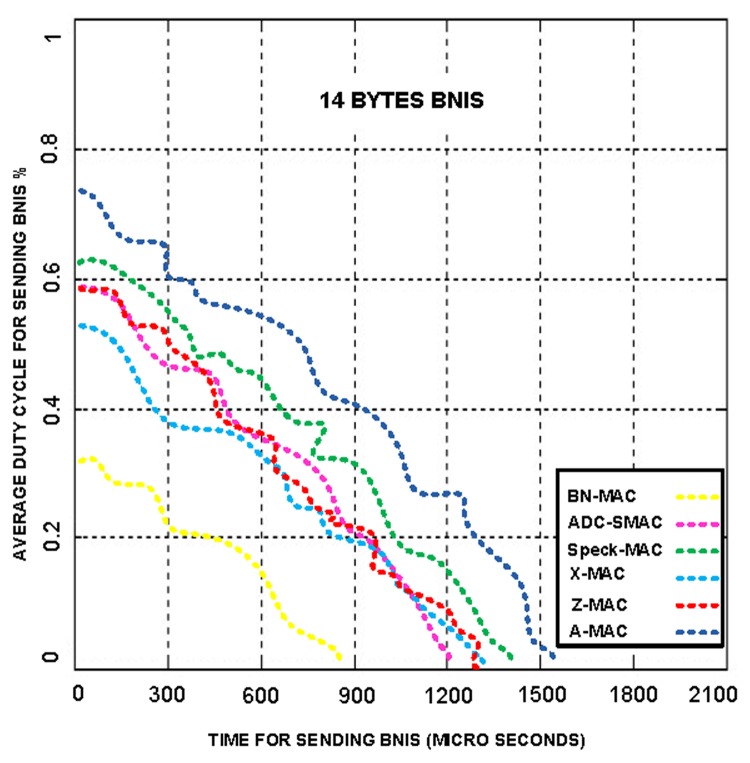
Time consumption for sending a BNIS message with BN-MAC and other MAC protocols.

The above model indicates that the energy consumption due to idle listening can be avoided. In BN-MAC, each node remains in the sleep state until the next data-sending schedule begins. The BN also announces its schedule; therefore, there is no probability of consuming energy. There is also no hidden terminal in BN-MAC. BN-MAC requires 832 μs to send a 14-byte BNIS message that produces a 0.3% duty cycle; other hybrid MAC protocols require an average of 1,209–1,532 μs to send each BNIS message that produces an average of 0.52–0.78 duty cycles, as shown in Figure 13. Thus, BN-MAC reduces the overhead by using fewer BNIS control messages and a synchronization message.

## 7. Intelligent Decision-Making (IDM) Model

We use the IDM model to increase the efficiency of BN-MAC. This model decides the nature of the environment, *i.e.*, whether the environment is indoor or outdoor. The IDM model forces the sensor nodes [41] to work on either the passive or active mode of communication in response to the nature of the environment. The IDM model helps to reduce energy consumption in both modes but particularly in the passive mode. The sensor nodes working in the passive mode do not consume the energy of their battery but may instead harvest energy, such as solar energy from the environment.

Let us assume that “K” is the number of sensor nodes available in the WSN, which are deployed to detect the presence or absence of the indoor and outdoor environment (IOE). K sensor nodes collect information regarding the IOE and then determine the nature of the environment. We have set values for the IOEs.

If D_i_ ≥ 1 indicates the presence of an indoor environment (IE), then D_i_ < 1 indicates the presence of an outdoor environment (OE). To prove “IE” and “OE”, we also use a third environment, *i.e.*, an unknown environment (UE). The detection process is based on the maximized probability of detection (MPD) method used by the Neyman-Pearson Lemma [42].

The K sensor nodes start the detection process from the UE because they are initially unaware of the nature of the environment. We set the probability of “UE” and pick a random variable that denotes the constraint of the optimized problem in the form of *UE* = α (*alpha*), as shown in Equation (16):

(16) E(UE)=α

One of the requirements for statistical optimization is establishing an expected value of UE. Hence, we maximize the expected value of the probability to detect UE with respect to the constraints of the expected value of the probability:

(17) $$\alpha =\sum_{k=0}^{\infty }\text{PK}\;(\text{K}=k){\text{UE}}^{k}(\beta^{k})$$

Substitute the value of α (*alpha*) in Equation (16) to find the UE that will be easier for the sensor node to sense out of IE and OE.

(18) $$E(UE)=\sum_{k=0}^{\infty }\text{PK}\;(\text{K}=\text{k}){\text{UE}}^{k}(\beta^{k})$$

UE*^k^* and (β*^k^*) are linked by the relative operating characteristics (ROCs) that are required to determine UE (ROCs are a strong method to validate probability estimates and particularly to compare its performance with deterministic estimate. it is two-dimensional process). We use the following probabilities to detect UEs and IEs:

UE_i_ = P (D_i_ < 1|IOE outdoor), βi = P (D_i_ < 1|IOE outdoor), and PD_i_ = P (D_i_ > 1|IOE indoor), γ_i_ = P (D_i_ > 1|IOE indoor).

Let us assume that the sensor nodes detect the environment independently. Thus, K sensor nodes detect UE based on the set probability values:

(19) $$\text{Di}=\text{IOE}-\sum_{k=0}^{\infty }\text{PK}\;(\text{K}=\text{k})\text{f}({\text{UE}}^{k}(\beta^{k}))$$

Di =1 indicates that the passive mode is initiated and the K sensor nodes reduce energy consumption. If Di ≥ 1 or Di ≤ 2, then the environment is known, and the sensor nodes stop using the energy of the battery and activate the passive mode to obtain energy from the environment. This condition indicates the presence of an OE.

The reduction in energy consumed can be calculated as follows:

(20) $$\text{EN}(\text{X})=\sum_{i=0}^{N}\text{E}(\text{i})\gamma (\text{ai})$$

(21) $$\text{EN}(\text{Y})=\sum_{j=0}^{N}\text{E}(\text{j})\partial (\text{aj})$$

Let us assume that EN(X) and EN(Y) are the total energy saved by two different regions of the WSN. E(*i*) and E(*j*) indicate the energy saved by nodes i and j during transmission, respectively. Thus, we can define the total saved energy of the WSN using Equation (7):

(22) $$T_{\text{saved}}=\text{EN}(\text{X})=\sum_{i=0}^{N}\text{E}(\text{i})\gamma (\text{ai})+\text{EN}(\text{Y})=\sum_{j=0}^{N}\text{E}(\text{j})\partial (\text{aj})+,\ldots ,\text{EN}(\text{n})=\sum_{j=0}^{N}\text{E}(\text{n})\partial (\text{an})$$

where T_saved_ is the total amount of saved energy.

If Di < 1, the active mode is activated. If Di ≥ 0 or Di < 1, then the IE is active, and the sensor nodes use the battery and external energy. The amount of energy consumed is calculated using Equation (23):

(23) $$Di=\text{IOE}+\sum_{K=0}^{\infty }PK(k=k)fUE^{K}(\beta^{K})$$

Therefore, we measure the total saved and consumed energy of the WSNs using passive and active modes based on the OE and IE. We also prove the energy saved using the WSNs using Lemma 1.

### Lemma 1

Bluetooth-enabled sensor nodes follow the energy preservation process during the passive mode using the integration method.

Here, we present the numerical time integrators that allow energy P(e) to be preserved. We begin by assuming an x-point quadrature formula with nodes N_i_. The required weight of a_i_ is obtained through Lagrange basis polynomials in interruption as follows:

(24) $$\lim(\tau )=\prod_{j=1,j\neq i}^{x}\frac{\tau -Nj}{Ni-Nj},\text{ai}=\int_{0}^{1}\lim(\tau )d\tau$$

Let a_1_, a_2_, a_3_,…, a*_x_* be different real numbers (0 ≤ Ni ≤1) for which a_i_ ≠ for all i. Note that all the values for different real numbers cannot be equal to a*_i_*. We use the polynomial p(d_0_) for satisfying the degree:

(25) p(do)=xo

(26) $$\text{p}(\text{d}0+\text{Nej})=\text{A}(\text{p}(+\text{Nej})\int_{0}^{1}\frac{\delta y}{\delta x}\nabla S(\text{p}(\text{d}0+\tau \text{s})\text{dx}$$

The quadrature formula with nodes N*_i_* and weights a*_i_* decreases the integrator to a specific collection of methods. We use polynomial degree 2*_x_* − 1; thus, Gauss points Ni are equal to 0 and shifted with the Lagrange polynomial specific collection for A(*x*).

This formula treats arguments in A(*x*) and *∇S* (*x*) differently than a partitioned numerical method. The solution obtained with these methods depends on the specific factorization of the vector field.

If A(*x*) = A is a constant matrix, let (1, 1) be a Hamiltonian system (a dynamic system used for the mathematical formalism to define the evolution equations. It also provides the significant understanding about the dynamics, even if the preliminary problem's value cannot be solved systematically). Thus, the Hamiltonian system becomes an energy-saving integrator. This result demonstrates that the sensor nodes also consume a minimum amount of energy during the passive mood.

## 8. Simulation Setup and Analysis of Results

Real WSN environments use low-power radios because of their high asymmetrical communication range and stochastic link characteristics. Simulation results could be slightly different from realistic experimental results [43]. If we make simple assumptions regarding wireless radio propagation, then the simulation results could be significantly different from realistic wireless radio features and diverse transmission power. It is critical to select a simulator that produces results that are reasonably close to the real environment. Thus, for our experimental simulation setup, we use ns-2.35-RC7 because it produces results that are highly similar to real environments.

In our experiments, the WSN is disseminated into N regions to collect information more quickly. We have simulated different realistic mobility- and static-based scenarios. The main goal of the simulation is to evaluate BN-MAC and compare it with other hybrid protocols, including Z-MAC, A-MAC, Speck-MAC, ADC-SMAC, and LPRT-MAC.

The simulation scenarios consist of 105 nodes with a 30-m transmission range. The sensor nodes are uniformly and randomly placed in a geographical area of 300 × 300 m^2^. The area is divided into N number of 75 × 75 m^2^ regions. The initial energy of each sensor node is set to 40 J. The bandwidth of the nodes is 50 kb/s, and the maximum power consumption for each sensor node is set at 16 mW. The sensing mode is 12 mW. Each sensor is capable of broadcasting the data at a power intensity ranging from −20 to 12 dBm.

The total simulation time is 35 min, and the pause time is set to 30 s during phase initialization at the start of the simulation. During this phase, the BN is in the warm up phase, and the remaining sensor nodes are automatically in power-saving mode. The presented results are an average of 10 simulation runs. The simulation parameters are illustrated in Table 2.

### 8.1. Network Coverage Efficiency

We conducted several simulation tests from different perspectives but with a particular focus on the network coverage efficiency after deploying 1 to 105 sensor nodes.

**Figure 14.**
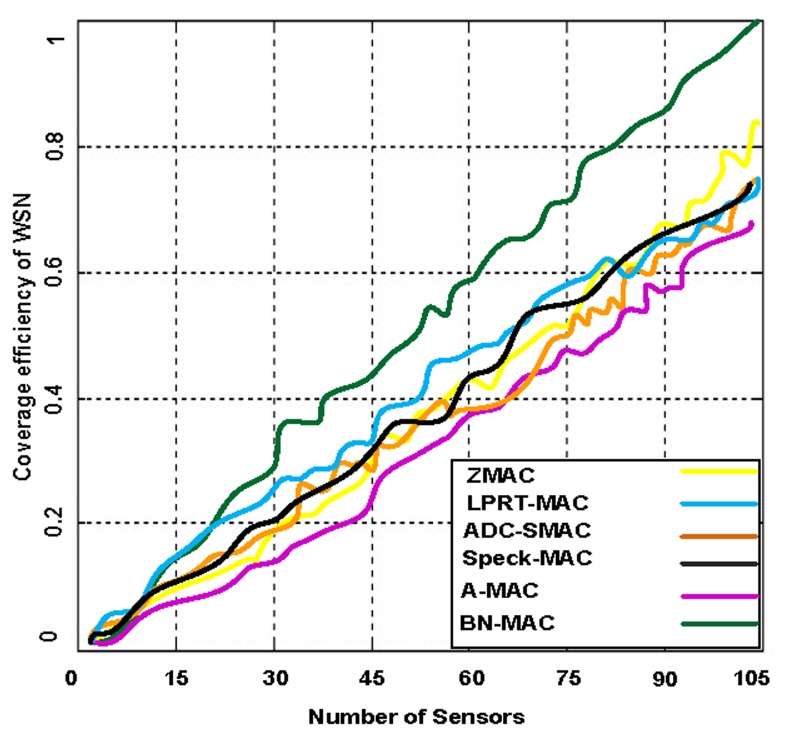
Coverage efficiency of the WSN using a different number of sensor nodes.

**Figure 15.**
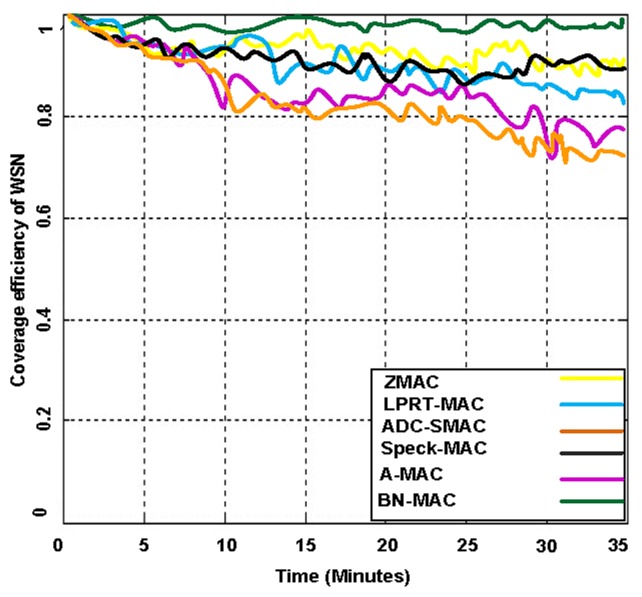
Coverage efficiency of the network at different intervals.

Network coverage can be regarded as how efficiently WSNs monitor the targeted area of interest. Network coverage can be considered a measure of the quality of service (QoS). Network coverage efficiency is measured in different ways depending on the nature of the applications and what is being monitored. The coverage is also crucial for maintaining the connectivity, which is defined as the capability of the sensor nodes to reach the base station. To measure the network coverage, we have created 15 sessions simultaneously to determine the actual behavior of the network using highly congested network scenarios. The simulations indicated that BN-MAC achieved a network coverage of 99.8%, whereas Z-MAC, A-MAC, Speck-MAC, ADC-SMAC, and LPRT-MAC achieve network coverage of 50%–87%, as shown in Figures 14 and 15.

BN-MAC achieves higher network coverage due to its compatibility with homogeneous sensor nodes (region nodes and BNs). The homogeneous set of nodes with a deterministic positioning attempts to guarantee the network coverage and connectivity with a minimum number of sensor nodes. The nodes are distributed in the targeted area of interest into regions to determine where to deploy the sensor nodes.

The limited energy resources must be used efficiently when choosing the BN because the BN is one of the major nodes in each region selected based on the presence of a high energy level using the LEI algorithm, which improves the connectivity of the WSN for a longer period of time. Furthermore, sensor nodes must be transitioned into the sleep mode using the AAS model while conserving energy to adjust the transmission range properly so that the sensor nodes may use the minimum amount of energy needed to communicate with the BN and neighbor nodes. The performance of BN-MAC is also improved because the one-hop neighbor node searches are optimized using LDSNS so that the data can be forwarded to the base station using the shortest and most efficient path. Energy is preserved by alleviating the routing load on some sensor nodes. By reducing the energy consumed via data routing, the network coverage is improved by prolonging the lifetimes of the sensor nodes. The minimum number of sensor nodes that are required to cover the entire network can be calculated as follows:

$${\text{N}}_{\min(\text{s})}=\frac{2\pi *A}{3\pi R^{2}}$$

where N_min(s)_ is the minimum number of sensor nodes required to cover the entire area to maintain connectivity and coverage and “r” is the sensing range of the sensor.

Let us assume that the sensing range is smaller than the dimensions of the monitoring area. $\frac{\text{Nmin}(\text{s})}{\text{Nmax}(\text{s})}$ is the maximum number of sensor nodes, and “*R*” is distance of the entire network.

### Lemma 2

$\frac{\text{Nmin}(\text{s})}{\text{Nmax}(\text{s})}$ is the upper bound on “*R*”, and N_min(s)_ is the lower bound on S_i_, where ${\text{N}}_{\min(\text{s})}=\frac{2\pi *A}{3\pi R^{2}}$.

**Proof:** Let the upper bound on “*R*” be linear, with the maximum number of sensor nodes (total number of sensor nodes) equal to N_max(s)_. The lower bound on “S_i_” is invariant with N_max(s)_. In addition, these bounds are not considered tight as long as they do not consider the transmission radius “T_r_” of the sensor nodes. However, a more accurate heuristic solution is required to follow these bounds closely regardless of changes that occur in the network parameters. Hence, the lifetime of the network is linearly asymptotic with N_max(s)_, and thus, “S_i_” will be constant with N_max(s)_.

**Figure 16.**
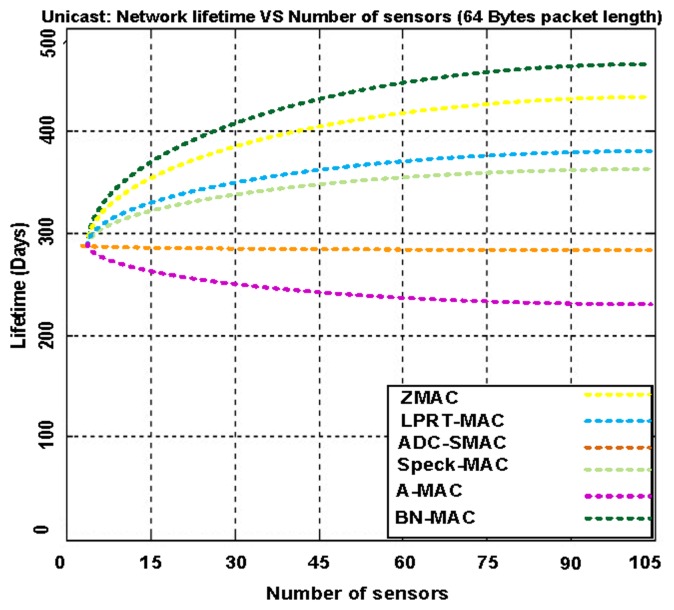
Lifetime of MAC protocols using a different number of sensors.

Figure 16 demonstrates the network lifetime using BN-MAC and the other hybrid MAC protocols. BN-MAC outperforms the other hybrid protocols because the other hybrid protocols are not capable of achieving the same network lifetime with an increased number of nodes. The network lifetime depends largely on the battery lifetime of the sensor node. The major concern is to extend the lifetime with respect to energy limitations. One way of extending the lifetime of the sensor nodes is to turn off redundant nodes and let the redundant nodes go into the sleep state to conserve energy. Our coverage-preserving BN idea reduces the energy consumption and therefore increases the system lifetime. BN-MAC has the ability to manage traffic and reduce the idle listening time. The BN-MAC mechanism consists of a semi-synchronous approach that helps to reduce the channel accessing time. BN-MAC also uses a short preamble message for accessing the channel without an integrated destination address in each preamble that reduces energy consumption and prolongs the network lifetime.

We illustrate the WSN lifetime using BN-MAC and competing hybrid MAC protocols as shown in Table 3.

**Figure 17.**
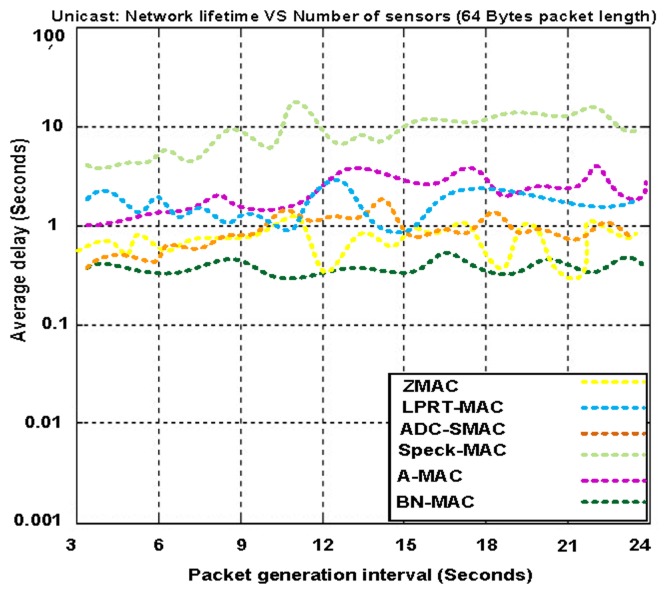
Average packet delay at different intervals.

Figure 17 presents the average packet delay at different packet generation rates using a fixed mobility of 1 m/s. The average delays of BN-MAC and Z-MAC are considerably less than those of A-MAC, ADC-SMAC, LPRT-MAC, and Speck-MAC. BN-MAC and Z-MAC have a low level of latency due to the use of a short preamble. The sensor nodes in BN-MAC use three directions (down, up, and local) to transmit data to neighbors according to whether the nodes are 1-hop closer, 1-hop farther, or at the same hop distance, respectively. When a sensor node has data to send, the sensor node first senses the channel to confirm whether the channel is free. If the channel is free, the sensor node transmits a short preamble message without a destination address because the destination address consumes the excess network bandwidth and reduces the network connectivity. We include transmission, propagation, and processing delays that help the preamble message to arrive at the required node during the channel polling time that also guarantees delivery of the data packets to the sensor node. The preamble transmission also overcomes the problem associated with small drifts in the clocks. Packet transmission starts when the transmission of the preamble ends. BN-MAC has automatic buffering because each node waits for the first packet to arrive, after which the remaining packets are buffered automatically to shorten the average packet delivery delay. The semi-synchronous mechanism is one of the most significant characteristics of BN-MAC because the semi-synchronous mechanism reduces the average packet delay.

**Figure 18.**
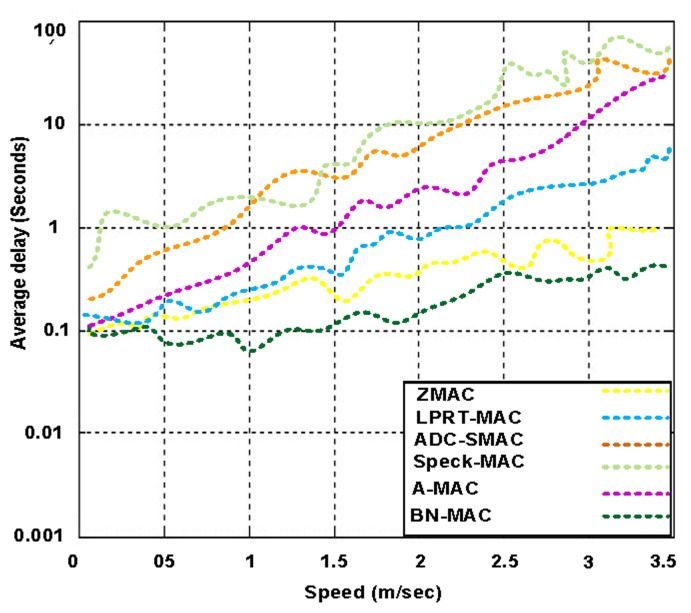
Average packet delay at different mobility levels (speed in m/s).

Figure 18 presents the average packet delay of BN-MAC and other participating protocols at different mobility rates. BN-MAC can manage its timeframe, number of random access frames, and rate of transfer frames while maintaining a nearly constant average delay. In contrast, Z-MAC (and other competing hybrid MAC protocols) does not have the mobility support, and thus, the average delay is increased. BN-MAC receives routing support from the EAP protocol at the network layer, which also helps to minimize the time needed for path discovery and route maintenance.

**Figure 19.**
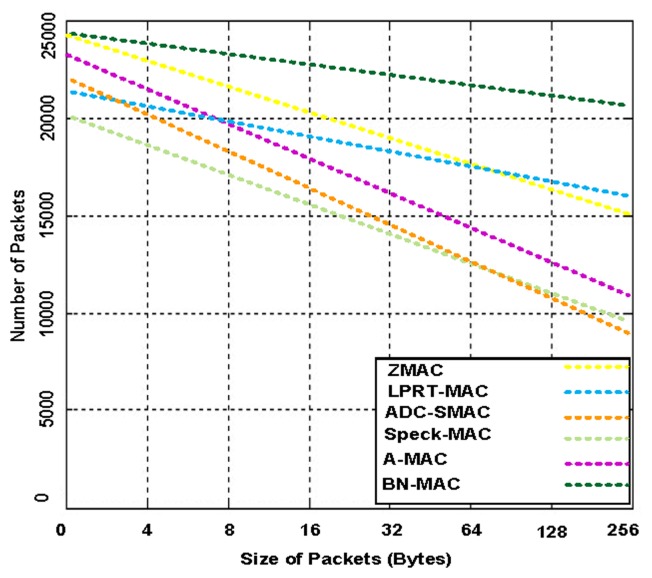
Number of packets delivered with variable packet sizes.

Figure 19 presents the number of packets delivered by BN-MAC and other protocols using variable packet sizes. BN-MAC delivers more packets than the other protocols. BN-MAC uses a balanced semi-synchronous schedule between the neighbor nodes. A semi-synchronous schedule helps to reduce energy consumption. Thus, the node energy exhibits a sharp decrease as the packet size exceeds an optimal length. This trend can be attributed to the maximum overhead, which increases the average re-transmission and thus decreases throughput. As the packet size increases, the exposed interval and probability of an interfering node increase. When BN-MAC uses 256 contention windows to avoid interfering nodes, there is a marginal likelihood that the packets will be dropped. In this manner, the size of the packets does not decrease the performance.

BN-MAC also uses the dynamic adjustment of packet size (DAPS) function, which handles the variable size of the packets. Thus, there is a marginal likelihood of packet re-transmission. BN-MAC is also advantageous in terms of sampling and randomization, thus avoiding the packet loss. The other MAC protocols use 1–16 contention windows for randomized listening before sending their preamble message. BN-MAC configures the contention window to 256 slots. Thus, there is small probability of dropping the data packet because only 5% of the nodes may choose the same slots at the same time.

**Figure 20.**
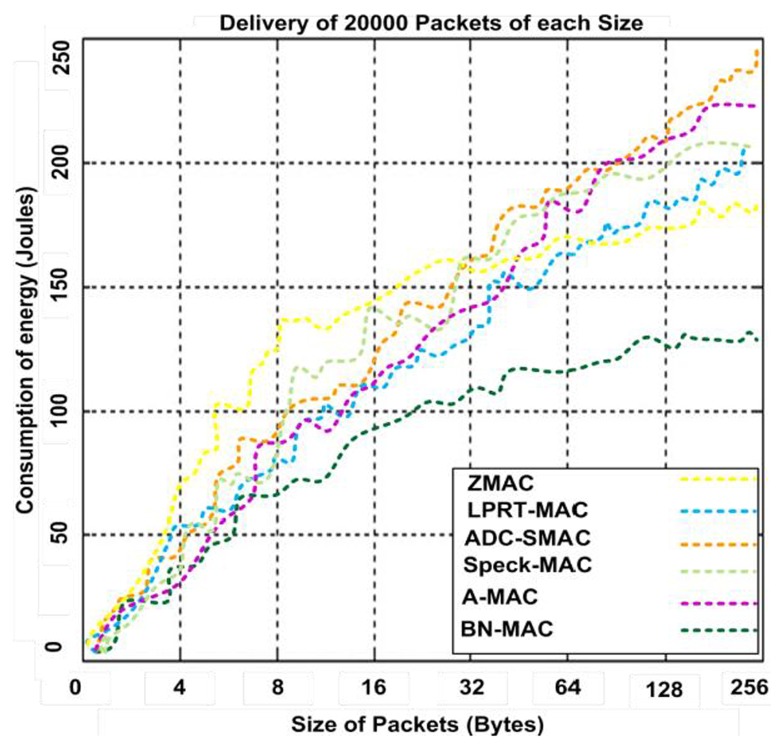
Energy consumption with variable packet sizes.

Figure 20 presents the energy consumption for 20,000 variable-length packets delivered and acknowledged using BN-MAC and other hybrid MAC protocols. BN-MAC consumes less energy than A-MAC, ADC-SMAC, LPRT-MAC, Speck-MAC, and Z-MAC. The variable size of the packets does not significantly affect BN-MAC due to the use of the DAPS function to handle the variable packet lengths. Less energy is consumed because the idle listening time is controlled, as the sensor node consumes the maximum amount of its energy without performing actions on the channel. The AAS model brings the sensor node into the sleep state after the event processes are no longer being monitored. Thus, the AAS model helps to maintain the fairness of the energy in the network during events.

**Figure 21.**
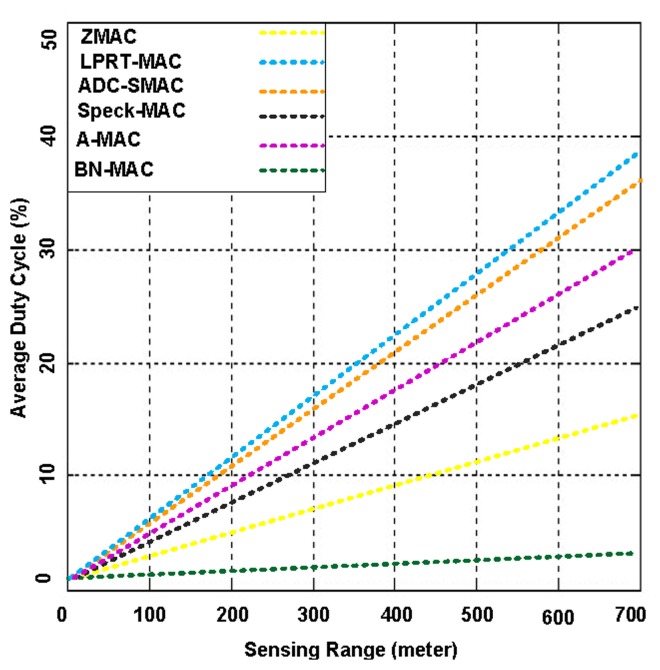
Average duty cycles at variable sensing ranges.

In Figure 21, we present the duty cycle for BN-MAC and other hybrid MAC protocols at different sensing ranges. BN-MAC exhibits a low duty cycle, whereas the other MAC protocols exhibit a higher duty cycle. The capacity to send packets at faster rate is affected as the sensing range is increased. In duty cycling, the node is periodically placed into the sleep state, which is effective for decreasing the energy dissipation in the network. In BN-MAC, energy is saved to bring the sensor node into the sleep state using the AAS model and a semi-synchronous technique. The packet adjustment-based duty cycle feature of BN-MAC also effectively reduces energy consumption without significantly reducing throughput and increasing latency. Other participating MAC protocols take an even longer period of time to access the channel and deliver the packets, thus increasing the energy consumption. As a result, the sensor node consumes additional energy when sending larger control messages, which consume 40%–70% of the network bandwidth. Thus, there is not a sufficient amount of power remaining in the other MAC protocols to send data for longer distances. For example, when the sensing range is 700 m, the duty cycle of BN-MAC is approximately 11%–12%, whereas A-MAC, ADC-SMAC, LPRT-MAC, Speck-MAC, and Z-MAC have duty cycles of 20%–29% because it takes a longer period of time to access the channel and forward the data packets.

### 8.2. Broadcast Traffic

We evaluated the performance of BN-MAC and other hybrid MAC protocols under broadcast flood traffic. In this experiment, we measure the strength of the BN when floods are first sent to other regions.

**Figure 22.**
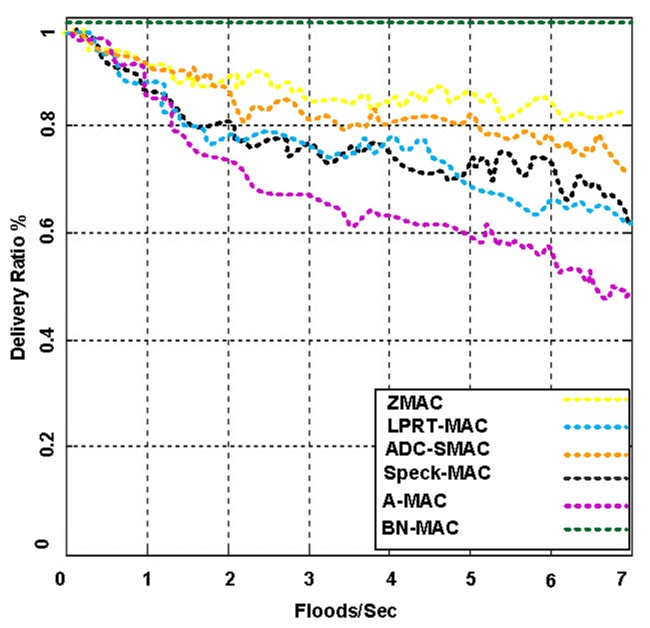
Delivery ratio under broadcast flood traffic.

Figure 22 presents the packet delivery rate for BN-MAC and the other competing MAC protocols under broadcast flood traffic. The packet delivery ratio of the BN is calculated as the total number of flood messages received from all nodes and delivered to other regions, which is divided by the total number of distinctive messages generated by all nodes. Each message of the node consists of a sequential number to find the uniqueness of the message. The simulation demonstrated that BN-MAC outperforms all of the other hybrid MAC protocols.

The BN-MAC curve is considerably higher than those for the other curves because the delivery ratio remains stable with the different network traffic floods. The high delivery rate is maintained because latency and idle listening are controlled. BN-MAC outperforms the other MAC protocols in high-traffic conditions.

BN-MAC also avoids network congestion using a congestion window with 256 slots, whereas A-MAC, ADC-SMAC, and LPRT-MAC do not have the ability to support simultaneous transmission, thus causing collisions. As a result, the same message is re-transmitted multiple times, and the packet delivery rate is reduced dramatically.

**Figure 23.**
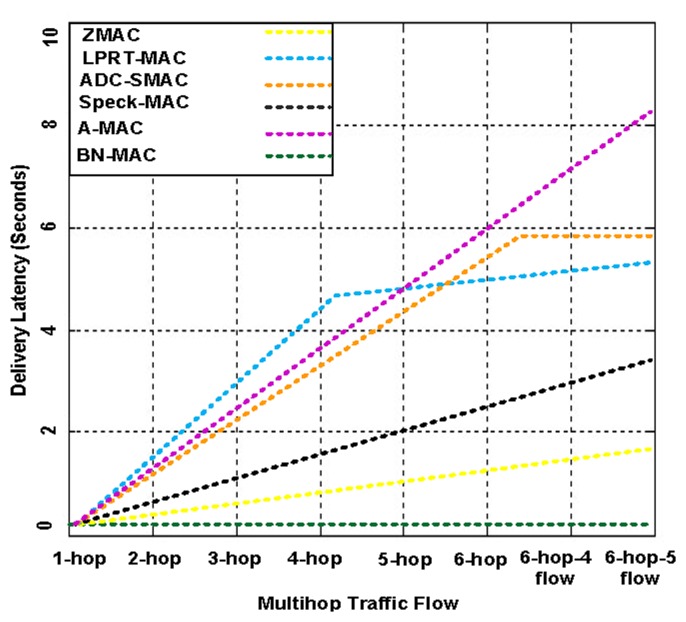
Latency of BN-MAC and the other hybrid protocols using different hops and traffic flows.

Figure 23 presents the latency of BN-MAC and the other hybrid protocols at different hops and traffic flows. BN-MAC provides uniform latency at various hops and different numbers of flows. The BN-MAC mechanism uses Anycast for scheduling and unicast for sending data at the one-hop neighbor node, which helps to improve the throughput and reduces the latency. There is also an extremely small probability of failure of a one-hop path. If a one-hop path fails, then a second alternative best one-hop path is chosen for intraregional data communication based on the stored information for the one-hop neighbor nodes. The mechanisms of the other MAC protocols support a multi-hop path technique. If one path fails, then it is difficult to immediately regain a path. Thus, the latency is increased, and the throughput decreases. Overall, BN-MAC has a low latency and outperforms the other hybrid MAC protocols.

### 8.3. Minimum Path Detection Time for Efficient Route

Routing in WSNs is usually assorted due to several limited constraints. The network performance depends on flexibility of routing protocol. From other side, an effective energy-efficient routing protocol design is big challenge for energy-constrained network [44]. In this experiment, our aim to choose proper routing protocol from existing routing protocols that should be compatible with BN-MAC features to create robust WSN. The suitability of routing protocol generally depends on application requirements because routing protocols maintain and discover the routes in the network. The function of routing protocols extend network lifetime while maintaining the high-quality of connectivity and allowing the reliable communication between nodes.

The sensor nodes are not accessible in some conditions because they are either located on the unreachable points or undergrounded for sensing the events. Hence, immediate human access to those sensor nodes is not possible [45]. Therefore, routing protocols should be mobility aware to deal with WSN applications' node mobility, event mobility and sink mobility.

Let us identify the routing protocol that should be more suitable with BN-MAC. Hierarchal routing protocols (HRPs) categorize the nodes based on their functionality. Nodes are divided into groups or clusters, and head node is selected to coordinate with inside and outside of the cluster [46].The HRPs are proposed to increase network lifetime. However, HRPs are not using multi-hop communication. As a result, HRPs can be used with BN-MAC because BN-MAC mechanism supports single hop search. Attribute or data-centric based is another category of routing Protocols that is named as sensor protocols for information via negotiation (SPIN). These routing protocols distribute the information among the sensor nodes using energy-constrained efficiently [47]. The base of SPIN communication nodes depends on specific knowledge of application. SPIN allows sensor nodes to disseminate information using less energy resources efficiently. Four types of SPIN protocols are available: SPIN-EC and SPIN-PP are used for point-to-point network, and another SPIN-RL and SPIN-BC are appropriate for broadcast network traffic and also providing 1-hop destination search. SPIN-RL does not provide optimal route at 1-hop destination, but helps to improve the search capability. Additionally, the mechanism of data advertisement of SPIN-RL is not highly guaranteed for reliable delivery of data. Energy aware routing protocol (EAP) is the energy efficient that uses sub-optimal routes to enhance the network lifetime. In EAP, single efficient path is chosen from many multiple paths to preserve energy. EAP has also priority over directed diffusion routing protocol family because EAP improves network performance and saves energy 21.5% to 44% [48].

We choose EAP protocol based on its compatible features with BN-MAC. EAP works in combination with the LDSNS model to find optimized 1-hop shortest paths (the LDSNS model is used to choose the best efficient one-hop neighbor node to establish the path to the destination node. LDSNS reduces energy consumption while choosing an efficient route to path).

EAP helps to maintain resource awareness, and improves the network lifetime. EAP also possesses some hierarchal features, which can support to BN to coordinate with intra and inter data transmission efficiently. BN-MAC with EAP maintains data aggregation that helps BN to coordinate and communicate without any reservation over WSN.

**Figure 24.**
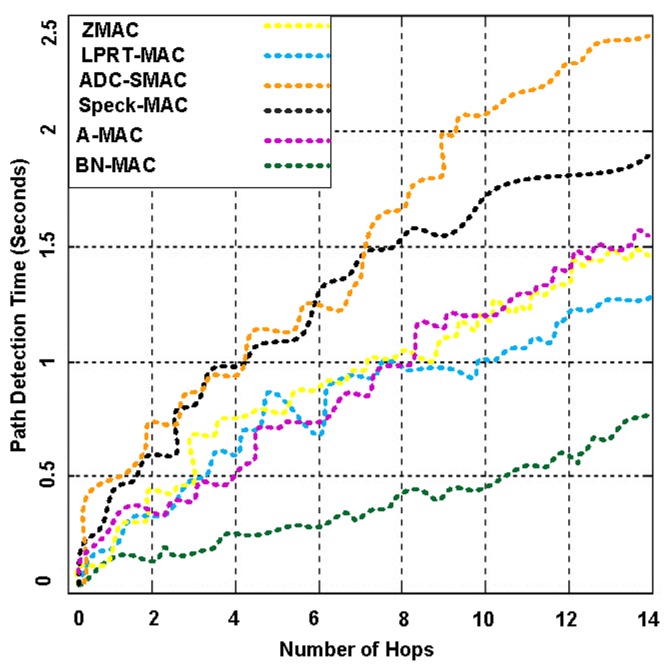
Path detection time for different number of hops.

In this experiment, we have used WSN consisting of 16 hop-destination with 15 concurrent established sessions. If, we analyze the Figure 24, it is observed that time for maximum hop number is calculated 0.8 s for BN-MAC whereas it takes from 2 to 3.5 s for other participating hybrid MAC protocols. The competing hybrid MAC protocols have used their original underlying routing protocols. The designed WSN for BN-MAC protocol is composed of regions. Each region consists of several sensor nodes that are controlled and coordinated by BN. In this experiment, BN broadcasts the control message while setting the paths for data transmission. The broadcasting message process consumes enough energy amount but sensor nodes lack the adequate energy resources. Thus, BN-MAC saves energy to use low duty cycle semi synchronous mechanism and AAS model, which also control the idle listening issue at the MAC level. From other side, EAP chooses single efficient path from group of multiple paths to save energy.

**Figure 25.**
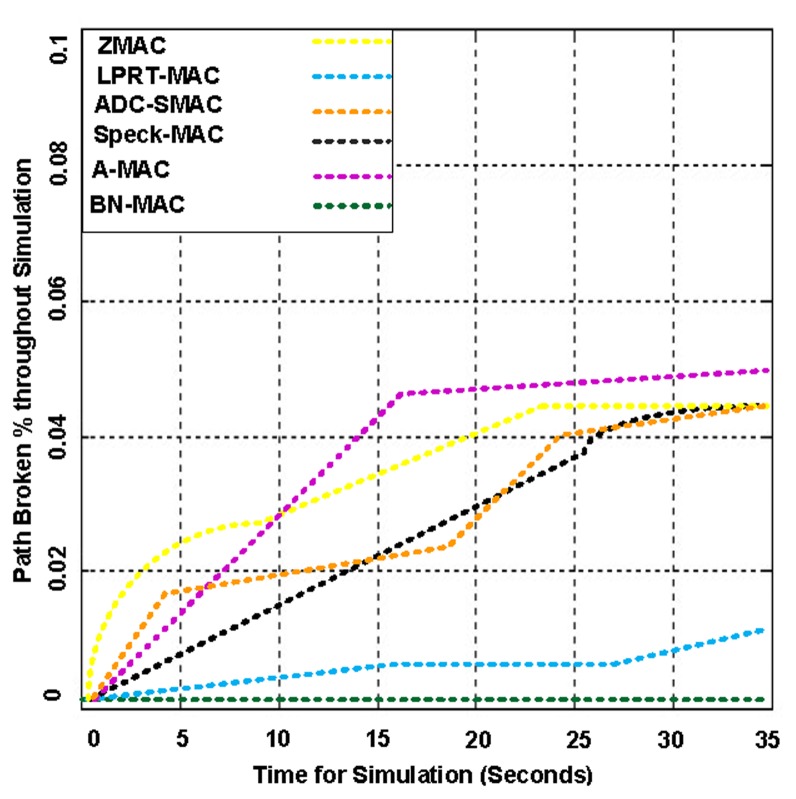
Broken paths% at different intervals.

Figure 25 shows broken routes during entire simulation time. BN-MAC is superior to competing hybrid MAC protocols throughout the entire simulation time. The competing MAC protocols experience the problem due to use of their original routing protocols. As a result, those protocols took enough time for route discovery. The route discovery time could be longer in some critical circumstances. Further, it is also easier to discover the route in WSNs based on single hop discovery process. The single-hop discovery process can handle the scalability and maintain the network mobility efficiently [37]. Path detection time for each hop is varied because it depends on the density of nodes that can be calculated as follows:

(27) $$\text{F}(\text{p})=\frac{H[Nr]}{2\pi r}$$

Let us assume that F(p) is the probability density function (a function that defines the comparative probability for the random variable to yield desired value; it is usually associated with absolutely continuous univariate distributions). H [Nr] is the number of hops in network and 2*πr* is the length of network. Thus, the value of *H*[*Nr*] can be calculated as follows:

(28) $$H[Nr]=\frac{4}{\pi }\arcsin(r)$$

where: “*r*”, distance from source node “*s*” to destination node “*d*”.

Substituting the value of *H*[*Nr*] and we get as:

(29) $$F(p)=\frac{\frac{4}{\pi }\arcsin(\text{r})}{2\pi r}$$

Simplifying (29), we get:

(30) $$F(p)=\frac{2\arcsin(\text{r})}{\pi^{2}r}$$

Determining the discovery time for broken links at each path, we need to consider the number of hops, network size and velocity of each node.

Where: *MH*(*t*), time for maximum number of hops; N(a), total network area and “*V_j_*” is corresponding velocity of each node.

Therefore, consumption time for maximum number of hops can be calculated as follows:

(31) $$MH(t)=\frac{\text{F}(\text{p})+\text{N}(\text{a})}{V_{j}}$$

Substitute the values of F(p) then we can get as follows:

(32) $$MH(t)=\frac{\frac{\frac{4}{\pi }\arcsin(\text{r})}{2\pi r}+\text{N}(\text{a})}{V_{j}}$$

(33) $$\text{N}(\text{a})=2\pi \int_{r+t/2}^{R}\lambda \;\text{xdx}$$

Substitute the value of N(a) in Equation (32) to get Equation (34):

(34) $$MH(t)=\frac{\frac{\frac{4}{\pi }\arcsin(\text{r})}{2\pi r}+2\pi \int_{r+\frac{t}{2}}^{R}\lambda \;\text{xdx}}{V_{j}}$$

## 9. Discussion of Results

Energy consumption has been known to be one of the greatest challenges of WSNs and will continue to be an immense challenge for the deployment of WSNs because the advancement in battery technology has been slower than the growth of processing power and data communication rates. This challenge has attracted researchers to introduce several new energy-efficient protocols to address this problem [48]. To address this challenge, several MAC protocols have been introduced at the MAC level. Hybrid MAC protocols are of paramount importance because they have lower energy consumption and better scalability than other categories of MAC protocols. In this section, we discuss and compare the strengths and weaknesses of BN-MAC versus other hybrid MAC protocols.

The Z-MAC protocol belongs to the hybrid family that supports multi-hop topology, and the nodes are fixed at their positions. The global time synchronization is used to synchronize the nodes, and slots are assigned to nodes but not fixed for each node. Z-MAC competes for the channel within any slot for data transmission. The assigned node is given high priority, which reduces collisions. The latency is increased, and the throughput is moderated. Z-MAC faces some problems because of the use of long preamble messages with a destination address, which increases the duty cycle and energy consumption. The fixed topology limits the node scalability of WSNs. The setup of the network phase becomes more difficult when a new node joins or leaves the network. Mobile nodes are unable to receive and send data packets. As a result, the network paths are broken.

Speck-MAC is a variant of the B-MAC protocol but exhibits redundant short-packet transmission and integrated destination addresses. Speck-MAC is efficient in transmitting the unicast messages, but the sender wastes excess energy by sending additional frames even though the receiver has already received the frames. The additional frames consume channel bandwidth and thus reduce the packet delivery rate. Speck-MAC supports mobility when the network path is broken, which increases latency.

LPRT-MAC is based on an efficient bandwidth allocation mechanism and uses super frames fixed into mini-slots to communicate with the base station. LPRT-MAC reduces the power consumption and coordinates with the channel. LPRT-MAC exhibits significant packet loss, which is affected by bit errors. LPRT-MAC also suffers from the star topology. Once the central node fails, the entire network suffers because the node maintenance requires a longer period of time in the WSN. This situation reduces the throughput and increases latency. Additionally, there is no dynamic node selection in LPRT-MAC, which could help to replace the node prior to its failure. The star topological network also exhibits low mobility because the nodes are tightly linked and cannot leave or join the network. LPRT-MAC also cannot be used for other communication topologies because it is not suitable for multi-hop WSNs due to topological constraints. A-MAC is based on a collision-free and non-overhearing mechanism and is particularly suitable for surveillance and monitoring applications. The nodes are attentive and inactive for longer periods of time until an event is detected. The major advantage of A-MAC is that it allows nodes to be notified in advance. However, A-MAC exhibits rather high idle listening and packet overhead. A-MAC consumes high amount of energy due to advertisements. Additionally, the high level of latency reduces the throughput. Sensor nodes are deployed tightly in A-MAC, causing mobility issues.

ADC-SMAC improves upon two features of S-MAC: node utilization and sleep delay. The advantage of ADC-SMAC is that it introduces flexible duty cycles and forwards new scheduling information to the neighbor sensor nodes. ADC-SMAC also supports real-time data communication. However, local synchronization in ADC-SMAC consumes a significant amount of energy and increases the latency. ADC-SMAC is not suitable for controlling idle listening and overhearing problems. ADC-SMAC also does not support mobility.

Our proposed BN-MAC is an energy efficient, semi-synchronous, and low-duty-cycle hybrid protocol that is especially designed to support applications in which events occur in different locations. BN-MAC is simulated on different region-based WSNs. Each region is controlled by a BN. BN-MAC does not compel any node to be elected as a BN based on a probability calculation. BN-MAC selects the BN based on the energy level that improves the network lifetime using the one-hop neighbor node with a semi-synchronous mechanism for scheduling at the MAC level. The multiple hops on the path create nonlinearities in the system. The node must wait for the next hop node to wake up. In this manner, the packet is held on every link of the path for different amounts of time [49].

EAR and LDSNS are used to determine the shortest efficient path at the routing level. Thus, there is a small probability of failure for the one-hop path. If the one-hop path fails, then the second best one-hop path is chosen based on the information stored for each one-hop neighbor node. Furthermore, BN-MAC performs localized time slot allocation without changing the time slots of existing nodes. This procedure reduces the latency and overhead and has a small probability of broken links. AAS is an energy-efficient search that reduces the energy consumption because the nodes automatically sense the environment. IDM is another feature implemented in BN-MAC. IDM forces the sensor node to work either in the passive or active mode, depending on the environment. Furthermore, the BN supports mobile environments because the sensor nodes exchange the schedule at the one-hop path but keep the information for two hops. This feature supports scalability. The characteristics of BN-MAC and other hybrid MAC protocols are illustrated in Table 4 that demonstrates the strengths and weakness based on simulation results.

## 10. Conclusions

This paper introduces a new energy-efficient BN-MAC hybrid protocol with mobility support. The BN-MAC is proposed and simulated for the battlefield scenario over WSNs. The protocol leverages the features from both CSMA and TDMA. CSMA features embedded in BN-MAC consist of semi-synchronization, which uses a short preamble to access the channel and maintain the schedule at the one-hop neighbor nodes. TDMA features are imported into BN-MAC for collision-free data delivery. We have introduced the IDM model, which automates the sensor nodes to work either in the passive or active mode with respect to the environment. The IDM model reduces the energy consumption when working in the passive mode. BN-MAC also has a reduced idle listening time based on the use of the AAS model. The AAS model forces the sensor nodes to go into the sleep state after collecting information on the events. Latency is reduced using the LDSNS model and EAP routing protocol. LDSNS provides the efficient one-hop path search. EAP is suitable for maintaining route discovery and path maintenance at the one-hop destination for faster data delivery. BN-MAC also uses two types of messaging schemes to control congestion and reduce latency: Anycast is used to obtain information from the one-hop neighbors, and unicast is used to forward the data. To evaluate the features of the proposed BN-MAC in the battlefield scenario, we used ns2.35-RC7 to demonstrate the performance from different perspectives. We have also simulated other hybrid protocols, such as Z-MAC, A-MAC, ADC-SMAC, LPRT-MAC, and Speck-MAC. The simulation results demonstrate that BN-MAC reduces the energy consumption by 18%–45%, improves the throughput, and decreases the latency compared with other hybrid MAC protocols with the same node density and topology. Our findings prove that BN-MAC is a scalable and mobility-aware protocol with real-time communication support. The protocol can be disseminated for other WSN applications, such as monitoring, controlling natural disasters, human-centric applications, and tracking mobile and static home automation devices. In the future, we will implement its features in a realistic environment.

## Figures and Tables

**Table 1.** Showing distribution of energy level of sensor node.

**Level of Energy**	**Voltage Level of Sensors**
Very High	3.3 to 3.7 V
High	3.0 to 3.3 V
High Moderate	2.7 to 3.0 V
Moderate	2.4 to 2.7 V
Low	2.1 to 2.4 V
Lowest	<2.0 V

**Table 2.** Parameter values used in the simulation WSN.

**Parameters**	**Description**
Transmission Range	30 m
sensor types	BT node sensors
Sensing Range of node	10 m
Initial energy of node	40 Joules
Bandwidth of node	50 Kb/S
Number of sensors	105 BT node rev-3
Size of network	300 × 300 square meters
Size of each region	75 × 75 square meters
Packet transmission rate	30 Packets/Sec
Data Packet size	4, 8, 16, 32, 64, 128, 256 bytes
Mobility model	Pheromone termite mobility model
Simulation time	35 min
Initial pause time	30 s
T_x_ energy	16 mW,
R_x_ energy	12 mW
Energy dissipation: actuation	0.022 mJ
Power intensity	−20 to 12 dBm
Minimum Cycle time, T	340 ms
Start time of BN-MAC	(0, 30) s
Sink location in each region	(60, 40)
MAC protocol	BN-MAC
Other MAC protocols	Z-MAC, A-MAC, Speck-MAC, ADC-SMAC, LPRT-MAC
Type of protocols	Hybrid protocols
Deployed models	IDM, LDSNS and AAS models
Mobility	0.5 to 3.5 m/s
Delivery of data at varying sensing range	100, 200, 300, 400, 500, 600 and 700 m
Routing Protocol	EAP

**Table 3.** Lifetime of hybrid MAC protocols over WSN.

**Name of MAC Protocol**	**No Traffic**	**Unicast Traffic (Intra Traffic)**	**Broadcast Traffic (Inter Traffic)**
ADC-SMAC	308	356	167
A-MAC	233	302	139
BN-MAC	387	493	268
LPRT-MAC	331	401	187
Speck-MAC	335	412	192
Z-MAC	344	437	197

**Table 4.** Characteristics and affecting factors of Hybrid Medium Access Control (MAC) Protocols and BN-MAC protocol.

**No:#**	**Parameter**	**ADC-SMAC**	**LPRT-MAC**	**BN-MAC**	**Speck-MAC**	**A-MAC**	**Z-MAC**
1	Coverage	Low	Medium	High	Medium	Low	Medium
2	Network lifetime	Low	Medium	High	Low	Low	Medium
3	Average Latency	Medium	Medium	Low	High	Medium	Low
4	Mobility	Low	Low	High	Low	Low	Medium
5	Throughput	Low	Medium	High	Low	Low	Medium
6	Residual energy	Low	Medium	High	Medium	Low	Medium
7	Packet size affect	High	Medium	Low	High	Medium	High
8	Duty cycle %	High	High	Low	High	Medium	Medium
9	Sensing Range-effect on performance	High	High	Low	Medium	Medium	Medium
10	Various floods/flows affect	Medium	High	Low	High	High	Medium
11	Delivery ratio %	Medium	Low	High	Low	Low	Medium
12	Path detection time	Hugh	Medium	Low	High	Medium	Medium
13	Broken paths%	Medium	Low	Low	Medium	High	High

## Notes

**Author Contributions**: This research work is part of Abdul Razaque's Ph.D. dissertation work. The work has been primarily conducted by Abdul Razaque under the supervision of Khaled M. Elleithy. Extensive discussions about the algorithms and techniques presented in this paper were carried between the two authors over the past year.

## Nomenclature

AAS: Automatic Active and Sleep
ACK: Acknowledgement
ADC-SMAC: Adaptive Duty Cycle SMAC
A-MAC: Advertisement-based MAC
BDIF: Broadcast Destinations Inter Frame
BN-MAC: Boarder Node Medium Access Control
BNIS: Boarder Node Indication Signal
BNVSP: Boarder Node Volunteer Selection Process
BSIF: Broadcast Source Inter Frame
BT node: Bluetooth- enabled Node
Ch-S: Channel Sampling
CD: Clock Drift
CDMA: Code Division Multiple Access
CP: Check Period
CTS: Clear-to-Send
CSMA: Carrier Sense Multiple Access
DAPS: Dynamic Adjustment of Packet Size
EAP: Energy Aware-Routing Protocol
EFB: Election Flag Bit
G-MAC: Gateway Medium Access Control
HRPs: Hierarchal Routing Protocols
IDM: Intelligence Decision Model
IE: Indoor Environment
IOE: Indoor and Outdoor Environment
LDSNS: Least Distance Smart Neighboring Search
LEI: Level of Energy Information
LPR-MAC: Low Power Real Time Medium Access Control
MAC: Medium Access Control
MPD: Maximized Probability Detection
OE: Outdoor Environment
ns2: Network Simulator-2
ROC: Relative Operating Characteristics
RTS: Request-to-Send
RX: Receiver
SF: Synchronized Frame
SP: Short Preamble
Speck-MAC: Speck-MAC
SPIN: Sensor Protocols for Information via Negotiation
SPIN-EC: SPIN via Negotiation Energy-Conservation
SPIN-BC: SPIN via Negotiation Broadcast Channel
SPIN-PP: SPIN via Negotiation Point-to-Point
SPIN-RL: SPIN via Negotiation Reliable Link
TX: Transmitter
UE: Unknown Environment
Z-MAC: Zebra Medium Access Control
